# Synthesis and Biological Evaluation of a Novel Dual-Targeting Small Molecule Drug Conjugate Modulating the Crosstalk between α5β1 Integrin and MDM2 in Glioblastoma

**DOI:** 10.1021/acsmedchemlett.5c00669

**Published:** 2026-01-19

**Authors:** Federico Arrigoni, Ana Ferrari, Helena Prpić, Elena Markeviciute, Alessia Muzi, Giuseppe Roscilli, Silvia Gazzola, Umberto Piarulli

**Affiliations:** † Department of Science and High Technology Università degli Studi dell’Insubria, Via Valleggio 11, Como 22100, Italy; ‡ A. Ferrari, A. Muzi, and Dr. G. Roscilli Takis s.r.l., Via Castel Romano 100, 00128 Rome, Italy

**Keywords:** dual-targeting SMDC, MDM2 inhibitor, p53, α5β1 integrin, cross-talk, glioblastoma, drug conjugate

## Abstract

Negative crosstalk between α5β1 integrin and the p53-MDM2 regulatory axis contributes to glioblastoma progression and therapeutic resistance. To explore the potential of dual inhibition of these two biological targets, the dual targeting small molecule drug conjugate (SMDC) (**1**) was designed by coupling the MDM2 inhibitor SAR405838 to a selective α5β1 integrin ligand *cyclo*(phg-*iso*DGR-*k*) (**7**) through a stable chemical linker. The resulting conjugate retained antiproliferative activity in U87-MG glioblastoma cells and induced p53 reactivation with minimal MDM2 induction. Cell cycle distribution analysis revealed a redistribution of cells from the G0/G1 phase to the G2/M phase exclusively upon treatment with conjugate **1**, suggesting that a different mechanism of action is engaged. These findings support the potential of this dual-targeting approach through a dual-targeting SMDC as a promising therapeutic strategy against high-grade glioma overexpressing the α5β1 integrin receptor.

## Graphical abstract

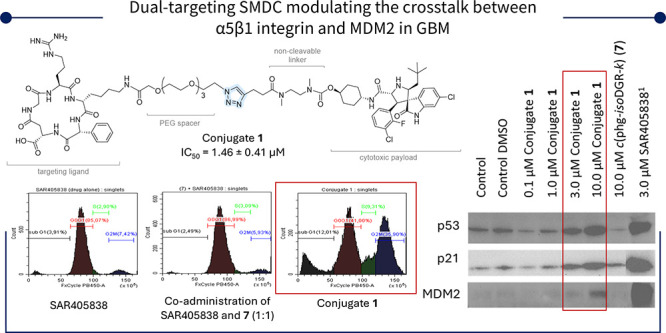

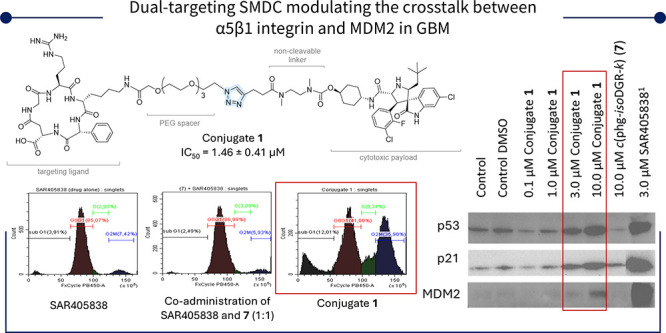

Glioblastoma multiforme (GBM) remains one of the most challenging brain tumors due to its aggressiveness and intrinsic resistance to standard therapies.^1^ Besides the alkylating agent Temozolomide (TMZ), which represents the first-line treatment,^2^ the pharmacological pipeline for glioblastoma is extremely limited, with only a handful of additional agents showing modest benefits.^3^ The lack of effective treatments contributes to the poor prognosis of GBM, with the average survival of approximately 5 years.^4^ The difficulty in treating GBM stems from its intrinsically heterogeneous nature and its ability to develop resistance. In this context, one of the mechanisms of resistance in GBM is the alteration of O6-methylguanine DNA methyl transferase (MGMT) activity, an enzyme responsible for repairing TMZ-induced lesions by removing methyl groups from the O6 position of guanine.^5^ Notably, earlier studies have demonstrated that increased intracellular levels of the tumor suppressor p53 can repress MGMT expression.^5,6^ The tumor suppressor p53, also known as the “guardian of the human genome”, is involved in a variety of biological pathways, such as apoptosis, cell cycle arrest, DNA repair, and autophagy, often related to suppressing aberrant cells.^7,8^ Due to many transcriptional activities, in tumors the p53 functions are often downregulated by the overexpression of a p53-negative regulator, mouse double minute protein (MDM2, also known in humans as HDM2), an E3 ligase that binds the N-terminal domain of p53, and through nuclear export, promotes its 26S proteasomal degradation.^9,10^

Notably, more than 85% of glioblastoma cases involve alterations affecting the p53 pathway.^11,12^ For these reasons, the development of small molecules targeting the MDM2 protein, thus impairing the MDM2-p53 complex formation and subsequent p53 degradation, has emerged as a promising strategy to restore p53 tumor-suppressor functions in GBM expressing p53.^13^ After the elucidation of the p53-MDM2 crystal structure (PDB ID: 4HFZ),^14^ numerous MDM2 inhibitors have been rationally designed to mimic the interactions between p53 and the three key hydrophobic residues of MDM2 (Phe19, Trp23, and Leu26).^15^ One of the early successful classes of p53-MDM2 inhibitors was *cis*-imidazoline small molecules Nutlin-3a (Figure 1), which causes cell cycle arrest predominantly at the G0/G1 phase^16^ and induces p53-dependent apoptosis in GBM and other cancer cell lines and xenograft models.^16,17^ Other classes of next-generation p53-MDM2 inhibitors include stapled peptides (ATSP-7041, ALRN-6924),^18,19^ benzodiazepine-2,5-diones (e.g., BDP),^20^ piperidinones (e.g., AMG232),^21^ spiro-oxindoles (e.g., SAR405838, APG115, BI907828)^22,23^ and β-carbolines (e.g., CPI-7c, SP141).^24,25^ In 2016, Verreault and co-workers^13^ reported the preclinical efficacy of the MDM2 inhibitor RG7112 in GBM, demonstrating induction of tumor cell death, reduced tumor growth, and increased survival. More recently, the use of the *cis*-imidazoline derivative RG7388 (Idasanutlin)^26^ and the piperidone AMG232 ^27^ has further highlighted the potential clinical relevance of MDM2 inhibition strategies in the treatment of glioblastoma. However, despite the extensive exploration of MDM2-p53 interactions, no FDA-approved MDM2 inhibitors are currently available, mainly due to p53’s pleiotropic functions and the resulting off-target effects.^28^ In addition, activation of p53 expression also leads to an increase in MDM2 levels, as a result of the p53-MDM2 negative feedback loop, in which p53 promotes the transcription of MDM2, potentially attenuating the overall antitumor efficacy of such inhibitors.^29^ A decreased survival of GBM patients has also been associated with a high expression of the α5 subunit of the α5β1 integrin receptor.^30^ Specifically, preclinical studies have demonstrated that this integrin contributes to key GBM hallmarks, including enhanced cell survival, migratory capacity, therapy resistance, and promotion of neo-angiogenesis.^31–33^ Remarkably, a “negative” crosstalk (in terms of downregulation and expression) between α5β1 and p53 pathway was proposed by Dontenwill and co-workers^34,35^ as a significant contribution to the chemoresistance in GBM.^36^ Indeed, by promoting the p53 activation with Nutlin 3a and inhibiting the integrin, a significant enhancement of the cell apoptosis process was observed. In a parallel approach, Marinelli and co-workers^37^ investigated the simultaneous targeting of α5β1 integrin and MDM2 protein in glioblastoma multiforme using a dual-targeting small molecule. Their results showed that the simultaneous dual inhibition enhanced cell cycle arrest and reduced proliferation of the U87MG glioblastoma cancer cell line, while also markedly decreasing cell invasiveness compared to treatment with Nutlin-3a alone.

**Figure 1:**
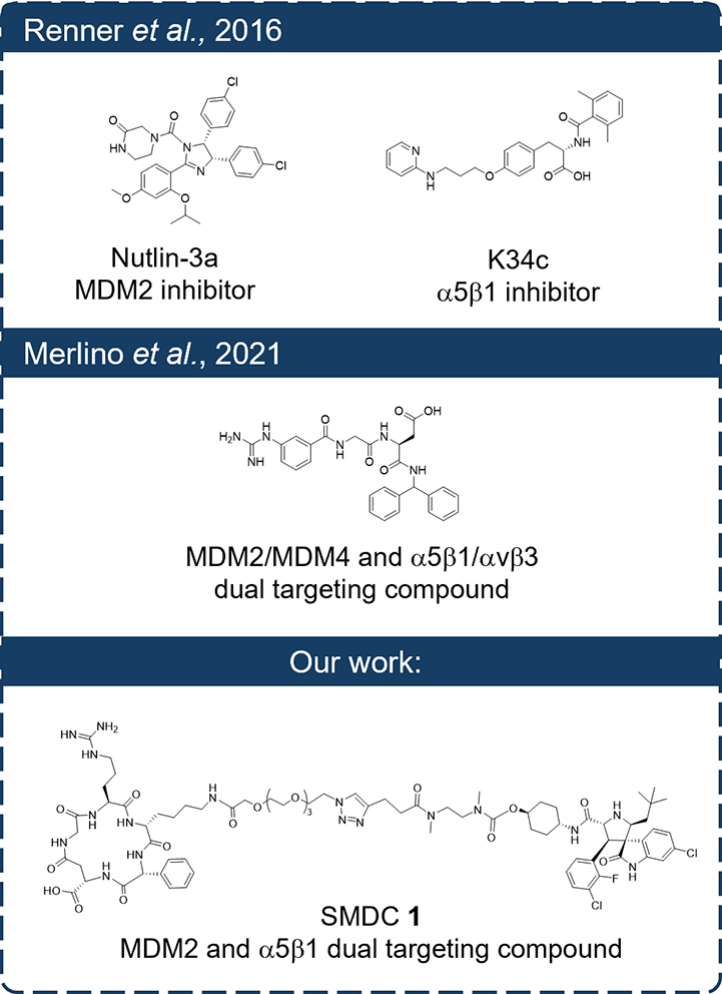
Summary of key studies targeting α5β1 and p53 pathways in GBM. Renner et al.^35^ used coadministration of α5β1 antagonists^36^ and Nutlin-3a; Merlino et al.^37^ developed the first dual-targeting molecule; our work introduces the first small-molecule drug conjugate **1** (SMDC) linking an α5β1 ligand to an MDM2 inhibitor.

Considering our general interest in the synthesis and biological evaluation of integrin ligand–based small-molecule drug conjugates (SMDCs) for the selective delivery of cytotoxic agents to cancer cells,^38–41^ and given the therapeutic relevance of both α5β1 integrin and MDM2 as cancer targets in GBM, we report herein the synthesis and biological evaluation of a new dual targeting conjugate, combining the spiro-oxindole derivative SAR405838 ^22,23^ (a potent MDM2 inhibitor with subnanomolar binding affinity), and the α5β1 integrin ligand *cyclo*(phg-*iso*DGR-k),^42,43^ originally identified by Kessler and co-workers as a highly potent and selective α5β1 integrin ligand, connected through a stable chemical spacer as depicted in Figure 1. The latter was selected over other candidates due to the convenient conjugation site provided by the lysine side-chain amino group, as well as its recently demonstrated ability to recognize α5β1 integrin-overexpressing cells *in vivo*, as validated by nano-SPECT/CT imaging using ^99m^Tc labeling.^44,45^ To design the desired dual-inhibitor conjugate featuring a stable linker, we first examined how chemical modification of the MDM2 inhibitor SAR405838 influences its biological activity in the U87-MG cancer cell line, which expresses wild-type p53. To our knowledge, no previous studies have reported functionalization of SAR405838. We therefore targeted the secondary hydroxyl group on the cyclohexyl ring for derivatization, as this site is solvent-exposed according to the cocrystal structure of the human MDM2-SAR405838 complex (PDB: 5TRF, Figure 2).^46^

**Figure 2:**
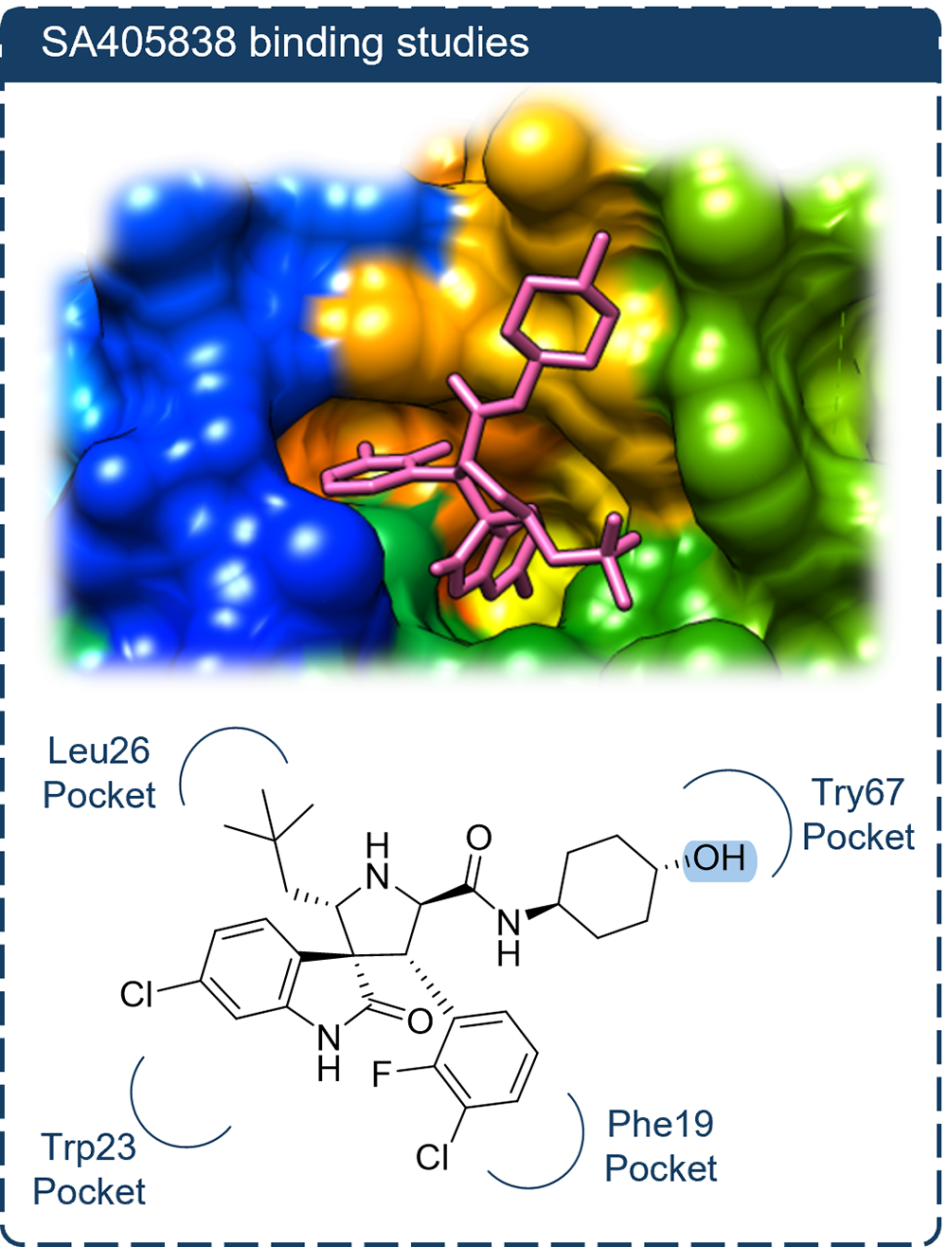
Predicted binding pose of SAR405838 in the MDM2 pocket (AutoDock Vina, based on PDB: 5TRF). The solvent-accessible hydroxyl group on the cyclohexyl ring is indicated as the conjugation site.

The choice of conjugation site was supported by our recent work, in which inserting a hydroxyalkyl chain at a solvent-exposed position of the β-carboline-based MDM2 inhibitor did not significantly impair the compound’s biological activity.^47^ To functionalize the selected hydroxyl group, we chose an *N*-methyl ethylenediamine–pentynoic acid linker (**4**) with a terminal alkyne for click reaction with the azide moiety and an amide-based backbone to avoid a hydrolyzable ester bond in the final conjugate. As outlined in Scheme 1, the secondary hydroxyl group of SAR405838 was first activated by conversion to the corresponding *p*-nitrophenyl carbonate **2** (69% yield). The intermediate was coupled under basic conditions with the previously synthesized alkyne linker **3**,^47^ affording the desired compound **4** in 72% yield. To evaluate whether linker installation affected biological activity, we tested compound **4** on U87-MG glioblastoma cells using the CellTiter-Glo luminescent cell viability assay after 72 h of incubation.

**Scheme 1:**
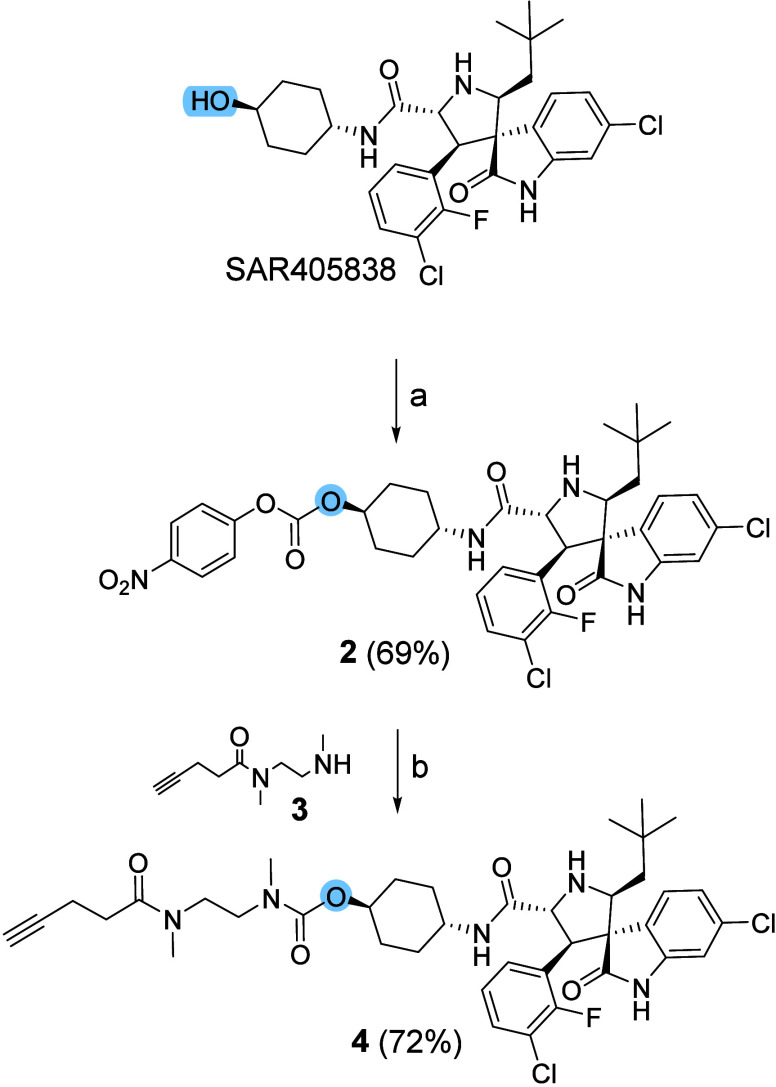
Synthesis of Derivative 4^a^,^b^

a Reagents and conditions: 4-nitrophenylchloroformate, pyridine, CH_*2*_Cl_2_, from −50 to −20 °C, 7 h.
b DMAP, iPr_2_NEt, CH_2_Cl_2_, 16 h.

The antiproliferative activity of **4** (0.76 ± 1.27 μM) was fully comparable to that of SAR405838 (2.01 ± 1.34 μM, respectively), thus confirming the suitability of the selected conjugation site (Figure 3). The previously identified α5β1 ligand, *cyclo*(*phg-iso*DGR-k) (**7**), was synthesized by slightly modifying a reported protocol by Kessler et al.^43^ The synthesis was initiated with the solid-phase peptide synthesis (SPPS) of Fmoc-*k*(Boc)-*phg*-*iso*D­(*t*-Bu)­GR­(Pbf)–OH (**5**) using DIC/Oxyma as coupling agents. After cleavage from 2-CTC resin using 20% HFIP in DCM, macrolactamization was performed using HATU/Oxyma/DIPEA^43^ under pseudodilution conditions (7.5 mM, peptide concentration), delivered via syringe pump, which afforded compound **6** in 72% yield. Obtained compound **6** was treated with cleavage cocktail TFA/EDT/TIS/water (95:2:2:1) to afford *cyclo*(phg-*iso*DGR-phg-*k*) (**7**) (Supp info, Synthesis 1.4. – 1.6.) which was then functionalized at the ε-amino group of lysine side chain with the azido-tetraethylene glycol spacer in 75% yield. The resulting intermediate **8** was subsequently coupled to compound **4** through the copper-catalyzed azide alkyne cycloaddition (CuAAC), affording the final conjugate **1** in 51% yield (Scheme 2).

**Figure 3:**
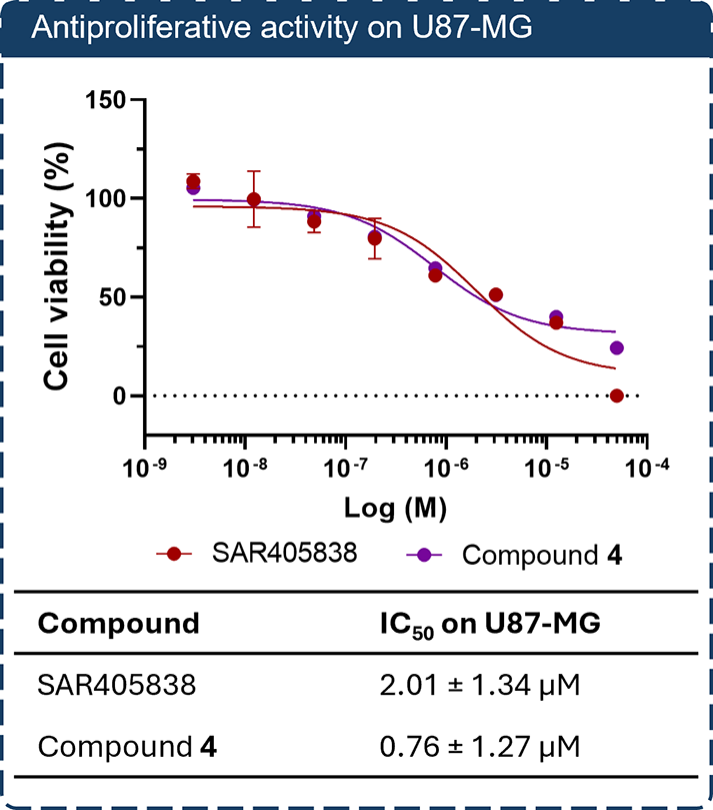
Antiproliferative activity of SAR405838 and compound **4** on U87-MG glioblastoma cells. Cells were treated with increasing concentrations of SAR405838 or compound **4** for 72 h, and cell viability was assessed using the CellTiter-Glo Luminescent Cell Viability Assay. Data represent mean ± SD of three technical replicates from one representative experiment out of at least three independent experiments, all yielding consistent results. IC_50_ values were calculated by nonlinear regression using GraphPad Prism; reported standard deviations reflect the fitting error of the regression model.

**Scheme 2:**
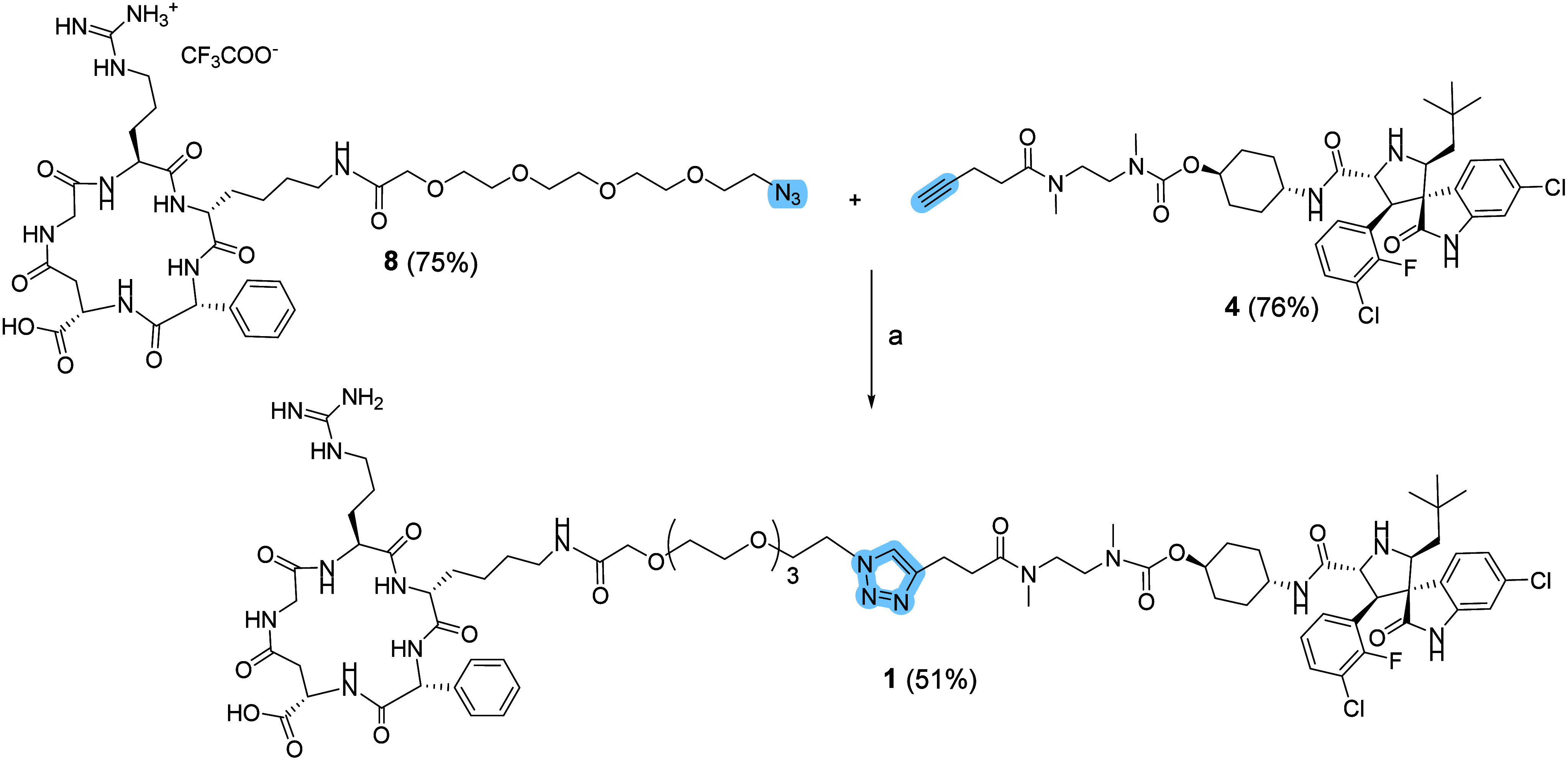
CuAAC of SMDC 1^a^

a Reagents and conditions: Compound **8** (1.1 equiv), compound **4** (1 equiv), CuSO_4_ × 5H_2_O (0.5 equiv), sodium ascorbate (0.6 equiv), N_2_, DMF:H_*2*_O = 1:1, 0–25 °C, 16 h.

The cytotoxicity of conjugate **1** was evaluated in the U87-MG cell line alongside the α5β1 integrin ligand **7**, SAR405838, and a 1:1 coadministration of **7** and SAR405838. Prior to these studies, the high expression of the α5β1 integrin receptor on our targeted U87-MG cancer cell line was confirmed by flow cytometry (FACS) analysis (Table S2, Figure S1 in Supp info). Interestingly, SMDC **1** displayed cytotoxicity comparable to that of the free drug SAR405838 (IC_50_ = 1.46 ± 0.41 μM vs 2.0 ± 1.34 μM) with no apparent loss of potency. Similar activity was observed for the coadministration of **7** and SAR405838, while integrin ligand alone (**7**) displayed negligible cytotoxicity (Figure 4).

**Figure 4:**
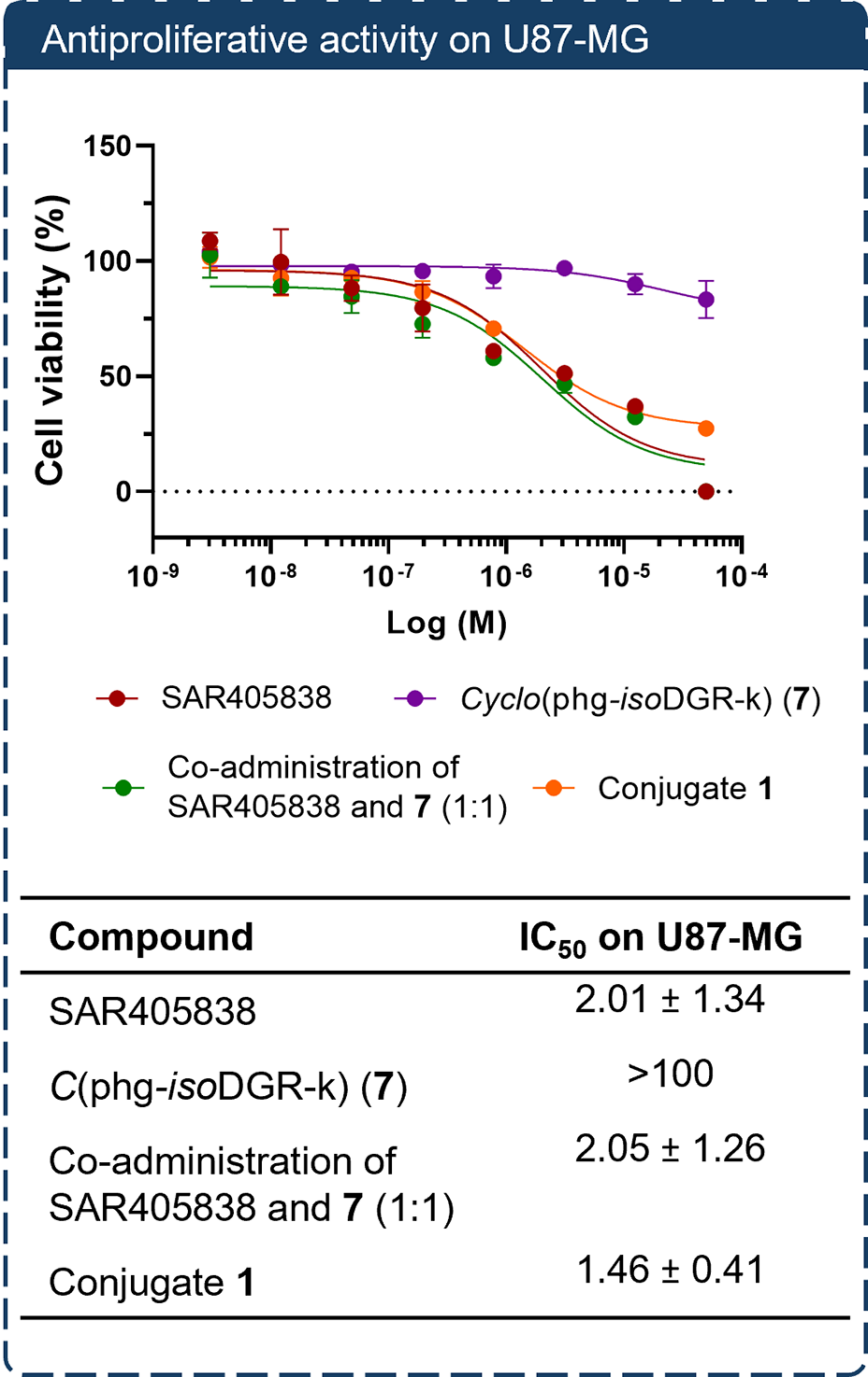
Antiproliferative activity of SAR405838, *cyclo*(phg-*iso*DGR-k) (**7**), their coadministration, and conjugate **1** in U87-MG glioblastoma cells. Cells were treated for 72 h with increasing concentrations of the indicated compounds, and viability was assessed using the CellTiter-Glo assay. Data represent mean ± SD of three technical replicates from one representative experiment out of at least three independent replicates, all showing consistent trends. IC_50_ values were calculated by nonlinear regression using GraphPad Prism; reported standard deviations reflect the fitting error of the regression model.

Subsequently, we interrogated the effect of the compounds on the expression of p53 and its associated proteins p21 and MDM2 in U87-MG cells by Western blot. Treatment with conjugate **1** for 24 h caused a dose-dependent induction of p53 (0.1, 1, 3, 10 μM), along with upregulation of p21. Notably, while SAR405838 at 3 μM robustly induced MDM2 via the canonical p53-MDM2 negative feedback (as expected for MDM2 inhibitors such as Nutlin-3a), conjugate **1** markedly attenuated MDM2 induction at the same concentration (Figure 5a). At 10 μM of **1** a slight increment of the expression of MDM2 is observed, which might reasonably rise by the activation of parallel regulatory pathways that upregulate MDM2 under highly stressed conditions.^48^ Nevertheless, while SAR405838 alone confirms the classical MDM2 inhibitor action (p53 stabilization + MDM2 feedback), the conjugate **1** demonstrates an ability to modulate that regulatory feedback (Figure 5a). This pattern suggests that conjugate **1** may partially disconnect p53 activation from its negative feedback control, sustaining a more robust p53/p21 response, which can reasonably be attributed to a synergistic effect arising from the simultaneous targeting of both α5β1 integrin and MDM2.^34,35^ To gain deeper insight into these results, we evaluated the effects of conjugate **1** on cell cycle distribution using FACS analysis. According to literature reports, in U87-MG cells expressing wt-p53, the MDM2 inhibitors, such as Nutlin-3a, induce cell cycle arrest predominantly in the G0/G1 phase.^16^ Consistent with the published data, treatment of the U87-MG cells with 10 μM of SAR405838, whose effect on this cell line has not been previously reported, resulted in a modest increase in the apoptotic sub-G_1_ fraction (3.91% vs 1.60% in control), which nonetheless corresponds to a measurable cytotoxic effect in the longer-term proliferation assay (Figure 5b, histogram 1). In contrast, 10 μM conjugate **1** induced a substantially higher sub-G_1_ peak (12.01%), indicating enhanced apoptosis (Figure 5b, histogram 4).

**Figure 5:**
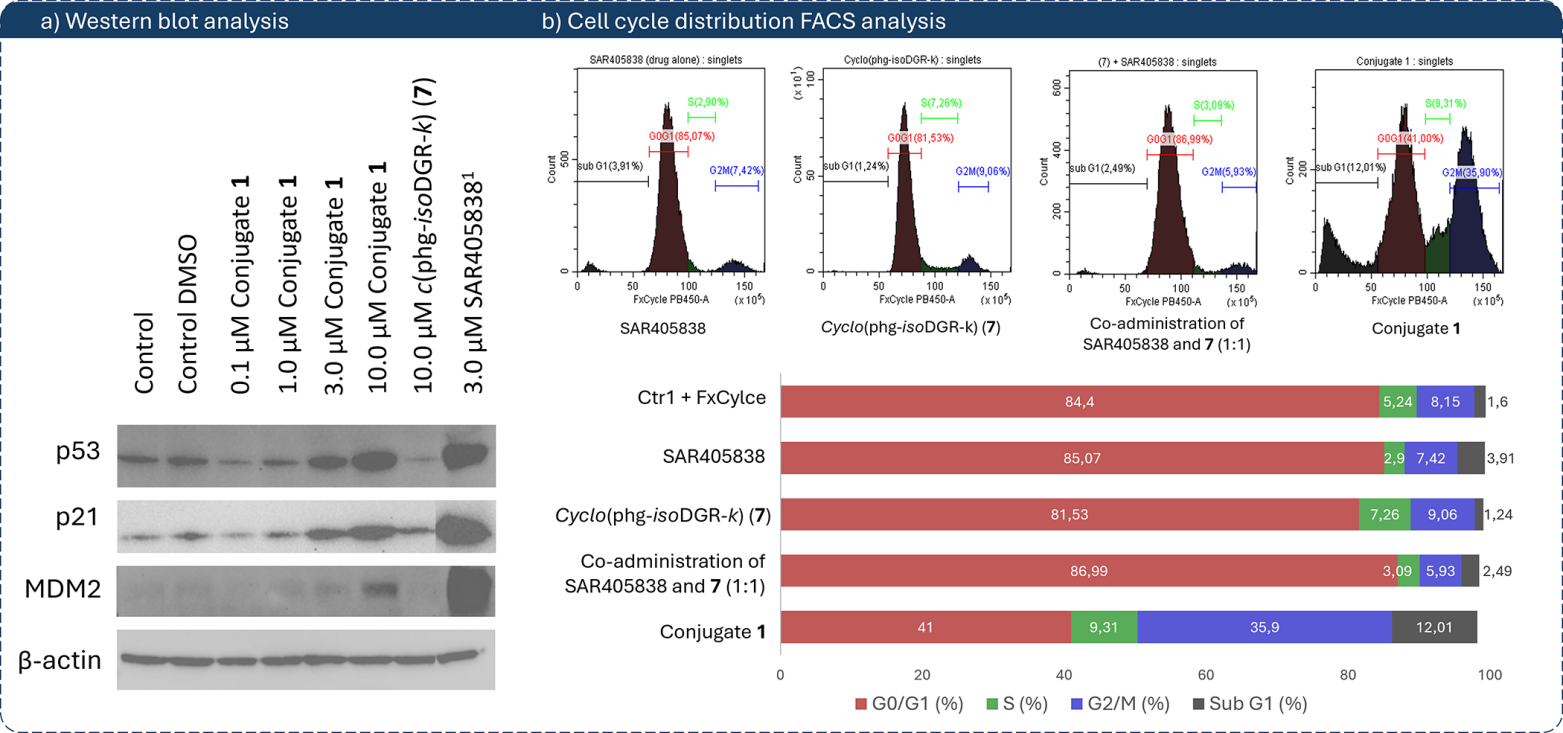
(a) Western blot response-dependent analysis of conjugate **1**, *cyclo*(phg*-iso*DGR*-k*) (**7**), and SAR405838 on p53, p21, and MDM2 expression in U87-MG cells. Concentration of SAR405838 chosen on previously performed dose-dependent Western blot analysis. (b) Cell cycle distribution analysis in U87-MG cells treated with 10 μM of the respective compounds for 24 h. DNA content was assessed by FxCycle staining and analyzed by FACS; histograms shown represent SAR405838 (histogram 1), *cyclo*(phg-*iso*DGR-*k*) (**7**, histogram 2), 1:1 molar coadministration of **7** and SAR405838 (histogram 3), and compound **1** (histogram 4). Cell population distributions in G0/G1, S, G2/M, and sub-G1 phases are summarized in the bar graph. Full gating strategy, replicate histograms, and control conditions are available in the Supporting Information (Table S3, Figure S2).

Conjugate **1** also drove a prominent accumulation in G_2_/M (35.90%), whereas SAR405838 alone showed only about 7.4% in G_2_/M, a percentage similar to control, indicating limited engagement of that checkpoint, as expected. With SAR405838 alone, we did not observe strong accumulation in G_0_/G_1_ relative to control as might be expected for MDM2 inhibitors: this may be because the G_0_/G_1_ arrest induced by p53 activation is transient; by the 24 h measurement point, some cells may already have progressed into apoptosis (sub-G_1_), thereby masking an earlier G_0_/G_1_ signature. Interestingly, glioma cells such as U87MG are particularly prone to p53-driven,^16^ reinforcing the fact that in our data we observed predominantly sub-G_1_ increase rather than robust G_0_/G_1_ arrest. Co-administration of SAR405838 and compound **7** (1:1) produced a profile close to SAR405838 indicating that most of the effect was attributed to the MDM2 inhibitor. This shift in the cell cycle profile suggests that conjugate **1**, by simultaneously inhibiting the overexpressed α5β1 integrin receptor and the MDM2–p53 complex formation, may influence cell cycle regulation through mechanisms not observed with either component alone or in coadministration. These effects may involve activation of stress signaling or DNA damage response pathways,^49^ resulting in a distinct cellular response compared to that of SAR405838.

In conclusion, we have developed conjugate **1**, a dual-targeted small-molecule drug conjugate that links the MDM2–p53 inhibitor SAR405838 to the α5β1 integrin ligand (**7**) through a stable linker, with the aim of investigating a possible synergistic effect arising by the modulation of the crosstalk between α5β1 integrin and MDM2 in glioblastoma by integrin–MDM2 crosstalk. Importantly, conjugate **1** retains potent antiproliferative activity in U87-MG glioblastoma cells, showing no loss of efficacy compared to the unconjugated MDM2 inhibitor (**1**). Western blot data confirms reactivation of p53 and its transcriptional downstream target p21, while concurrently attenuating the compensatory upregulation of MDM2. The cell cycle analysis further reveals a distinct arrest profile relative to compound SAR405838 alone, with increased accumulation in the G2/M and sub-G1 phases. Although further work is needed to elucidate the mechanism of action of conjugate **1**, as well as to assess its selectivity toward α5β1 overexpressing cancer lines, our findings support the dual-targeting approach and provide the foundation for further development of precision therapeutics against glioblastoma.

## Supplementary Material



## Notes

The PDF file includes general procedure for the chemical synthesis of the compounds, NMR spectra, HPLC purity analysis, HRMS/LC–MS spectra, and all the procedures of the performed biological assays, including data of α5β1 integrin expression levels on different cancer cell lines and FACS analysis data (PDF)

## #

Deutsches Krebsforschungszentrum (DKFZ), Im Neuenheimer Feld 280, 69120 Heidelberg, Germany

## Abbreviations

GBM: glioblastoma multiforme
TMZ: temozolomide
MGMT: O6-methylguanine DNA methyl transferase
MDM2: mouse double minute protein
SMDC: small molecule drug conjugate
DMAP: dimethylaminopyridine
SD: standard deviation
DMF: dimethylformamide
DMSO: dimethyl sulfoxide
