# Supplementary material for: Synthesis and Biological Evaluation of a Novel Dual-Targeting Small Molecule Drug Conjugate Modulating the Crosstalk between α5β1 Integrin and MDM2 in Glioblastoma
Source: ACS Med Chem Lett. 2026 Jan 19;17(2):450–7. doi: 10.1021/acsmedchemlett.5c00669 (PMC12907918; doi:10.1021/acsmedchemlett.5c00669)
Supplement: Supplementary file 1 [file ml5c00669_si_001.pdf]

Supporting Information

Synthesis and Biological Evaluation of a Novel Dual-Targeting Small Molecule Drug Conjugate Modulating the Crosstalk between α5β1 Integrin and MDM2 in Glioblastoma

Federico Arrigoni,[a‡] Ana Ferrari,[b‡] Helena Prpić,[a] Elena Markeviciute,[a] Alessia Muzi,[b] Giuseppe Roscilli,[b*] Silvia Gazzola[a*] and Umberto Piarulli[a*]

[a] F. Arrigoni, H. Prpić, E. Markeviciute, S. Gazzola and U. Piarulli Department of Science and High Technology Università degli Studi dell'Insubria Via Valleggio 11, Como, 22100, Italy

[b] A. Ferrari, A. Muzi, and Dr. G. Roscilli Takis s.r.l., Via Castel Romano 100, 00128 Rome, Italy

Email: roscilli@takisbiotech.it

E-mail: s.gazzola@uninsubria.it

E-mail: umberto.piarulli@uninsubria.it

Table of content

Materials and Methods 3

1. Chemical synthesis 3

2. Biological analysis 12

Analytical spectra 18

3. HPLC traces and HRMS spectra 18

4. NMR spectra 20

Supplementary references 26

Materials and Methods

1. Chemical synthesis

All commercially available reagents were utilized in their original form. Anhydrous solvents were obtained from commercial sources and extracted from the container using a syringe under slightly positive nitrogen pressure. Reaction progress was monitored by analytical thin-layer chromatography using pre-coated TLC sheets (ALUGRAM®Xtra SIL G/UV254) with a silica gel layer of 0.20 mm (60 with fluorescent indicator UV254). Compounds were visualized using UV fluorescence, aqueous Potassium Permanganate, Ninhydrin, Cerium Ammonium Molybdate, and Vanillin chromophores staining. Flash column chromatography was performed using Sigma-Aldrich technical-grade silica gel (pore size: 60 Å, 230-400 mesh; particle size: 40-63 μm).

^{1}H-NMR spectra were recorded on a spectrometer operating at 400.16 MHz, and ^{13}C-NMR spectra were recorded on a spectrometer operating at 100.63 MHz, with complete proton decoupling. Proton chemical shifts are reported in ppm (δ) with the solvent reference relative to tetramethyl silane (TMS), used as the internal standard. Spin multiplicities are described using the following abbreviations: s = singlet, d = doublet, t = triplet, q = quartet, m = multiplet, br = broad signal, dd = doublet of doublet, dt = doublet of triplet. Solid-phase UV detection was carried out using a SHIMADZU 2600i UV-Vis double-beam spectrophotometer.

HPLC purifications were performed on SHIMADZU LC-20AP and Waters 600 HPLC systems, utilizing a Sepachrom Robusta 100 Å C18 5 μm 250 mm x 21.2 mm column with a flow rate of 15 mL/min. HPLC traces of final products Mass spectra (MS) were acquired on a Waters MicroMass ZQ mass spectrometer equipped with an ESI ion source and a quadrupole detector, coupled with a Waters 600 HPLC system (column: SynergiTM 4 μm 150 x 4.6 mm; flow rate: 1 mL/min) or on Thermo Fisher Scientific Orbitrap Exploris 120 equipped with a Vanquish UHPLC System (Thermo Fisher Scientific) and a reversed-phase column AccucoreTM C18 50x2.1 mm, 2.6 μm (Thermo Fisher Scientific). For freeze-drying, the product was dissolved in water or a water/acetonitrile mixture (1:1) and frozen with dry ice. Freeze-drying was conducted for at least 48 hours at -50 °C using the Telstar-LyoQuest instrument**.**

The N_3-PEG4-CH_2COOH (CAS: 201467-81-4) was synthesized following established literature procedure, with analytical data consistent with previously published results.[1–3]

1.1. 2’-(4-Nitrophenoxycarbonyl)-SAR405838 (2)

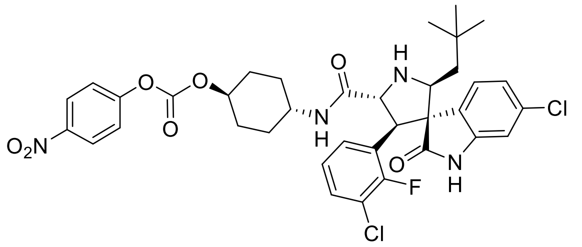

$C_{36}H_{37}Cl_2FN_4O_7$
MW = 727.71 g/mol

Commercially available SAR405838 (20 mg, 0.036mmol, 1equiv.) was dissolved in 400 μL of dry THF under N_2 atmosphere. Pyridine (17 μL, 0.216 mmol, 6 equiv.) was added dropwise, and the mixture was cooled down to -50°C. 4-nitrophenyl chloroformate (72 mg, 0.356 mmol, 10 equiv.) was added portion-wise over 3 hours, and the mixture was stirred at -20°C for 7 hours. The mixture was then diluted in EtOAc and washed with 1 M $KHSO_4$ (2 x 7 mL) and brine (2 x 7 mL). The organic phase was dried over Na_2SO_4 and concentrated under reduced pressure. The crude residue was purified by flash chromatography on silica gel (gradient: from Hex/EtOAc 1:1 to Hex/EtOAc 1:4), affording product **2** as a pale-yellow solid (18 mg, 69%, yield).

HRMS (ESI+): *m/z* calc. for $[C_{36}H_{37}Cl_2FN_4O_7 + H]^+$ = 727.2096, found: 727.2079.

1.2. *tert*-butyl methyl(2-(N-methylpent-4-ynamido) ethyl) carbamate (3)

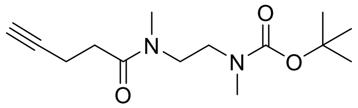

$C_{14}H_{24}N_2O_{23}$
MW = 268.38 g/mol

A solution of commercial 4-pentynoic acid (156 mg, 1.593 mmol, 3 eq) in dry DMF (4 mL) was cooled to 0 °C under a nitrogen atmosphere**.** DIPEA (430 μL, 2.655 mmol, 5 eq) was added, and the mixture was stirred for 10 min at 0 °C. A solution of COMU (100 mg, 0.531 mmol, 1 eq) in dry DMF (4 mL) was added, and the solution was stirred for 5 minutes at 0°C. A solution of *N*-(Boc)-*N, N'*-dimethyl ethylenediamine in dry DMF (2 mL) was added, and the reaction was allowed to reach room temperature and stirred for 2 hours. 1 mL of a solution of HOAt in DMF (0.6 M) was added, then the reaction was stirred overnight at room temperature. The mixture was diluted with an EtOAc/CH_2Cl_2 mixture (4:1, 100 mL) and washed with 1 M aqueous $KHSO_4$ (2 × 15 mL), a saturated aqueous $NaHCO_3$ solution (1 × 15 mL), and brine (1 × 20 mL). The organic phase was dried over Na_2SO_4 and concentrated. The solid was purified by flash chromatography (Hex/EtOAc 3:2) to afford **3** as a yellow oil (140 mg, 98%).

R_f = 0.46 (95:5, CH_2Cl_2/MeOH); **HRMS (ESI+)**: *m/z* calc. for $[C_{14}H_{24}N_2O_3 + H]^+$: 269.1859, found: 269.1859; **^{1}H NMR (400 MHz, CD_2Cl_2)** δ 3.47 – 3.42 (m, 2H), 3.32 (t, *J* = 6.1 Hz, 2H),

2.98 + 2.91 (s, rotamer A + B, 3H), 2.85 + 2.83 (s, rotamer B + A, 3H), 2.51 – 2.48 (m, 4H), 1.97 (s, 1H), 1.44 + 1.42 (s, rotamer A + B, 9H) ppm.

1.3. 4-pentynamido-SAR405838 (4)

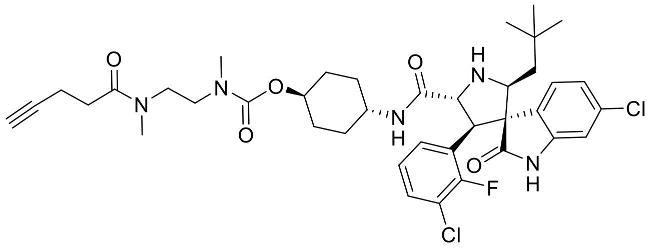

A solution of *tert*-butyl methyl(2-(N-methylpent-4-ynamido) ethyl) carbamate **3** (17.18 mg, 0.064 mmol, 3 equiv.) in dry CH_2Cl_2 (1.06 mL) was cooled to 0 °C under a N_2 atmosphere, and TFA (0.53 µL) was added. The reaction mixture was stirred for 2 h at room temperature, concentrated under reduced pressure, and excess TFA was removed with toluene. Diethyl ether was added to the residue, and the resulting suspension was evaporated under reduced pressure to yield the corresponding TFA salt. The crude product was used in the subsequent step without further purification.

Obtained crude was dissolved in dry DMF (600 µl) and cooled to 0°C under N_2 atmosphere, after which DIPEA (16.46 µl, 0.095 mmol, 4.5 equiv.) was added, followed by the addition of the prepared solution of 2'-(4-Nitrophenoxycarbonyl) - SAR405838 (**2**) (15.6 mg, 0.021 mmol, 1 equiv.) in 300 µL of dry DMF. To the resulting mixture, DMAP (2.82 mg, 0.023 mmol, 1.1 equiv.) was added, and the reaction was allowed to reach room temperature and stirred overnight (24 h). After completion, DMF was evaporated under reduced pressure. The crude product was purified by flash chromatography on a column using a DCM/MeOH gradient (99:1 to 9:1), affording **4** as a pale-yellow solid (12 mg, 76%).

$\mathbf{R_f}$ = 0.14 (95:5, CH_2Cl_2/MeOH); **HRMS (ESI+)**: *m/z* calc. for $[C_{39}H_{48}Cl_2FN_5O_5 + H]^+$ = 756.3089, found: 756.3070; **^{1}H NMR (400 MHz, Acetone-d$_6$)** δ 9.47 (s, 1H), 8.01 (s, 1H), 7.79 (d, *J* = 7.4 Hz, 1H), 7.70 (d, *J* = 7.5 Hz, 1H), 7.61 (t, *J* = 7.0 Hz, 1H), 7.51 (d, *J* = 8.0 Hz, 1H), 7.26 (t, *J* = 7.5 Hz, 1H), 7.16 – 7.02 (m, 2H), 6.78 (d, *J* = 1.4 Hz, 1H), 4.63 – 4.58 (m, 1H), 4.48 – 4.41 (m, 2H), 3.71 – 3.67 (m, 1H), 3.59 – 3.56 (m, 1H), 3.53– 3.50 (m, 2H), 3.46 – 3.38 (m, 2H), 3.06 (m, rotamer A, 4H), 2.93 – 2.86 (m, rotamer B, rotamer A+B, 4H), 2.60 – 2.50 (m, 2H), 2.45 – 2.41 (m, 2H), 1.97 – 1.90 (m, 4H), 1.49 – 1.43 (m, 2H), 1.39 – 1.29 (m, 3H), 0.95 – 0.81 (s, 10H) ppm; **^{13}C NMR (101 MHz, Acetone-d$_6$)** δ 178.07, 173.05, 172.97,

158.53, 156.07, 144.62, 134.31, 129.87, 129.51(conformer 1), 129.48(conformer 2), 128.45, 128.32, 127.32, 125.57, 125.49, 125.41, 122.75, 121.02, 120.82, 110.69, 69.98, 69.68, 67.22, 67.17, 66.22, 50.97, 48.18, 47.53, 43.97, 34.89, 34.81, 31.56, 31.47, 30.95, 30.46, 15.23, 14.91 ppm.

Solid-Phase Peptide Synthesis − General Procedure[4,5]

Peptide synthesis was carried out using the Fmoc strategy on 2-chlorotrityl chloride (2-CTC) resin (100-200 mesh, 1 % dvb, loading 1.2 – 1.47 mmol/g purchased from J&J Scientific).

Loading of the resin. The CTC resin (250-400 mg) was added to a 10 mL syringe equipped with a PP-frit and cannula. After swelling for 60 min in DMF with continuous agitation on an IKA KS shaker (640 rpm), the solvent was discarded. A solution of Fmoc-Arg(Pbf)-OH (5.0 equiv.), DIPEA (10 equiv.) in DMF (10 mL/g resin) was loaded and rotated at room temperature for 2 hours. Afterward, the solution was discarded, and the resin was thoroughly washed with a DMF/IPA cycle (5 x 1 min alternating solvents each time), followed by DMF (3 x 1 min) using 8 mL/g resin. Trityl chloride groups were capped with a solution of DCM, MeOH, DIPEA (8:1:1) at room temperature (3 x 15 min), followed by the washing procedure described. The resin loading was determined gravimetrically after drying with DCM (3 x) and then under vacuum for 2 days (Ls = 0.86 mmol/g resin).

Fmoc-deprotection. The resin-bound Fmoc-protected peptide underwent treatment with a solution of piperidine in DMF (20%, v/v, 3 x 10 min) and was washed with a DMF-IPA cycle (5 x 1 min alternating solvents each time), followed by DMF (3 x 1 min) using 8 mL/g resin.

Peptide coupling using DIC/Oxyma. A solution of Fmoc-AA-OH (5.0 equiv.), Oxyma (5.0 equiv.) and DIC (5.0 equiv.) in DMF were added to the resin-bound free amine peptide and rotated for 5h at rt. The solution was discarded, and the resin was washed with a DMF-IPA cycle (5 x 1 min, alternating solvents each time), then with DMF (3 x 1 min) using 8 mL/g resin. The resin was washed using the described procedure.

Cleavage of the resin. Before the final cleavage, the resin-bound peptide underwent thorough washing with a DMF-IPA cycle (5 x 1 min alternating solvents each time), DMF (3 x 1 min), and DCM (3 x 1 min) using 8 mL/g resin. A solution of HFIP in DCM (20%, v/v) was added to the resin-bound peptide, rotating for 2 hours (8 mL/g resin). The procedure was repeated three times, washed with DCM (3 x 8 mL). The obtained solutions were combined and concentrated under reduced pressure.

Analysis of the coupling. **LC-MS**. 2-3 resin beads were transferred to a separate vial, stirred with a few drops of cleavage solution for 10 min, and the solvents were removed under reduced pressure; the resulting crude peptide was analyzed by LC-MS. **Kaiser Test**. A few drops of ninhydrin (5% w/v) solution in EtOH, phenol solution (80% w/v in EtOH), and KCN solution (1 mM in pyridine) were added over some resin beads. The mixture was heated to 90 °C: a yellow color indicated the absence of free amines, while a blue-purple color indicated their presence.

General procedure for Fmoc deprotection: A 0.1 M solution of *N*-Fmoc-protected compound (1 equiv.) in DMF was cooled to 0°C under argon. Piperidine (5 equiv.) was added, and the reaction was stirred at room temperature for 2 h. Volatiles were evaporated under reduced pressure to give the crude deprotected compound.

1.4. (H)-k(Boc)-phg--*iso*D(O^{t}Bu)-G-R(Pbf)-OH (5)

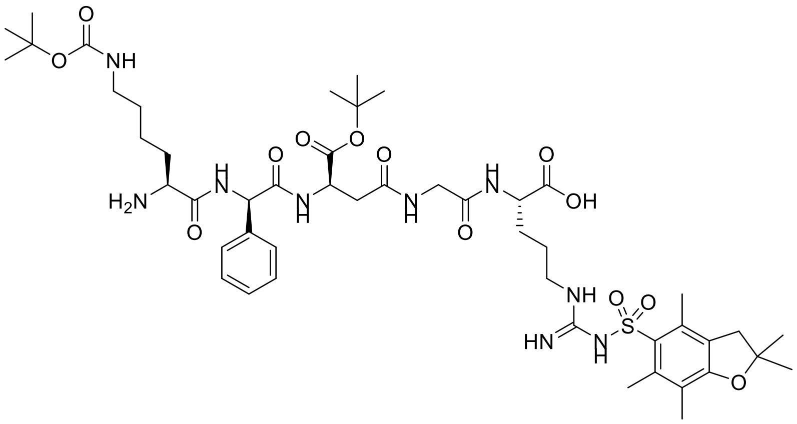

$C_{48}H_{73}N_9O_{13}S$
MW = 1016.22 g/mol

The pentapeptide (H)-k(Boc)-phg-*iso*D(OtBu)-G-R(Pbf)-OH was synthesized by solid phase utilizing the protocol described above, loading 400 mg of 2-CTC resin. Each coupling step employed DIC/Oxyma as coupling agents, with varying reaction times (as described in the table).

Fmoc-AA-OH	Moles (mol)	Equiv.	MW (g/mol)	Mass (g)	Time
**Fmoc-(Pbf)Arg-OH**	2.94	5	648.77	1.910	2h
**Fmoc-Gly-OH**	1.72	5	297.21	0.511	16h
**Fmoc-Asp(α-O*t*Bu)-OH**	1.72	5	411.25	0.707	16h
**Fmoc-D-(Ph)Gly-OH**	1.72	5	373.41	0.642	3h
**Fmoc-D-Lys(Boc)-OH**	1.72	5	468.55	0.806	3h

The amounts of DIC (1.72 mmol, 5 equiv., 264 μL) and Oxyma (1.72 mmol, 5 equiv., 245 mg) were used for each coupling. The obtained crude was then purified by RP-HPLC [Gradient (15 ml/min flux): 95% (H_2O + 10% CH_3CN) / 5% (CH_3CN + 10% H_2O + 0,1% HCOOH) for 3 min, then to 100% (CH_3CN + 10% H_2O + 0,1% HCOOH) in 15 min, t_R = 14.8 min, 30 min method], and freeze-dried affording **5** as a white powder (247 mg, 70%).

HRMS (ESI+): *m/z* calc. for $[C_{48}H_{73}N_9O_{13}S + H]^+$: 1016.5117, found: 1016.5121; **^{1}H NMR** (400 MHz, DMSO-d$_6$) δ 9.22 (s, 1H), 8.64 (d, *J* = 7.5 Hz, 1H), 8.25 (s, 2H), 7.81 (d, *J* = 7.3 Hz, 1H), 7.39 (d, *J* = 7.3 Hz, 2H), 7.29 (m, 3H), 7.03 (s, 1H), 6.73 (s, 1H), 6.56 (s, 1H), 5.54 (s, 1H), 4.39 (dd, *J* = 13.2, 7.0 Hz, 2H), 4.01 (dd, *J* = 12.6, 7.0 Hz, 2H), 3.66 (d, *J* = 5.4 Hz, 4H), 3.06 – 2.98 (m, 2H), 2.97 (s, 2H), 2.92 – 2.85 (m, 2H), 2.81 (d, *J* = 7.3 Hz, 2H), 2.65 (d, *J* = 5.4 Hz, 1H), 2.61 (d, *J* = 5.2 Hz, 1H), 2.59 – 2.53 (m, 1H), 2.48 (s, 3H), 2.43 (s, 3H), 2.01 (s, 3H), 1.67 (s, 2H), 1.61 – 1.44 (m, 2H), 1.41 (s, 6H), 1.37 (s, 9H), 1.30 (s, 9H), 1.09 (m, 1H) ppm; **^{13}C NMR (101 MHz, DMSO-d$_6$)** δ 173.78, 170.62, 169.64, 169.28, 169.18, 168.16, 157.40, 156.13, 155.52, 138.26, 137.22, 134.22, 131.38, 128.23, 127.51, 127.05, 124.28, 116.22, 86.24, 80.58, 77.33, 55.95, 52.93, 52.46, 49.98, 45.39, 42.45, 42.25, 36.65, 32.26, 29.30, 29.13, 28.28, 28.24, 27.42, 25.46, 21.99, 18.91, 17.55, 12.24 ppm.

1.5. *cyclo*(phg- *iso*Asp(O^{t}Bu)-Gly-Arg(Pbf)-*k*(Boc) (6)

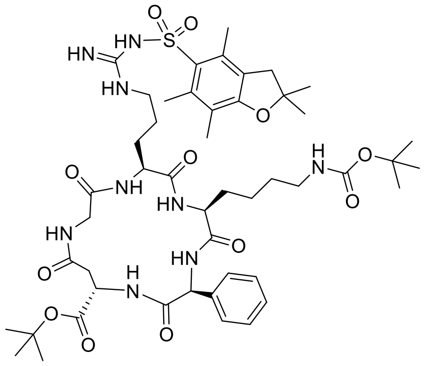

$C_{48}H_{71}N_9O_{12}S$
MW = 998.21 g/mol

To a dry DMF (4.2 mL) under N_2 atmosphere, DIPEA (110 μL, 0.63 mmol, 10 equiv.), HATU (2.29 mg, 0.006 mmol, 0.1 equiv.) and OXIMA (0.90 mg, 0.006 mmol, 0.1 equiv.) were added. Linear peptide NH_2-*k*(Boc)-phg-*iso*Asp(O^{t}Bu)-Gly-Arg(Pbf)-COOH) (**5**) (64 mg, 0.063 mmol, 1 equiv.) was dissolved in dry DMF (4.2 mL) and taken into a syringe A. Syringe B was charged with the remaining amount of HATU (69.5 mg, 0.183 mmol, 2.9 equiv.) and Oxyma (26.0 mg, 0.183 mmol, 2.9 equiv.) in dry DMF (4.2 mL). The syringes were connected to the flask and placed on the automatic syringe pump. Solutions in syringes A and B were added simultaneously at a flow rate of 1.5 mL/h. After the reagent addition reaction was stirred for

11 h (24 h altogether). After LC-MS confirmed the formation of the product with the desired mass, the reaction was stopped and concentrated under reduced pressure. The residue was added dropwise to the falcons containing cold Et_2O, inducing precipitation. The precipitate was centrifuged (3x), obtaining **6** as a white solid (45 mg, 72%).

LC-MS (ESI+): *m/z* calc. for $[C_{48}H_{71}N_9O_{12}S + H]^+$ = 999.50, found: 1000.07; **^{1}H NMR (400 MHz, Acetone-d6)** δ 8.49 (d, *J* = 9.1 Hz, 1H), 8.27 (s, 1H), 8.05 (s, 1H), 7.80 (s, 1H), 7.66 (d, *J* = 6.7 Hz, 2H), 7.43 – 7.34 (m, 3H), 7.28 (d, *J* = 6.4 Hz, 1H), 6.69 (s, 2H), 6.08 (s, 1H), 5.83 (d, *J* = 9.2 Hz, 1H), 4.57 (s, 1H), 4.49 (s, 1H), 4.41 (s, 1H), 4.23 – 4.19 (m, 1H), 4.11 – 4.04 (m, 2H), 3.79 – 3.76 (m, 1H), 3.56 (dd, *J* = 14.6, 7.3 Hz, 2H), 3.39 (s, 1H), 3.10 (s, 3H), 2.88 – 2.82 (m, 1H), 2.77 – 2.70 (m, 1H), 2.69 (s, 3H), 2.60 (s, 3H), 2.17 (s, 2H), 1.54 (s, 6H), 1.49 (s, 9H), 1.47 (s, 9H), 1.28 (t, *J* = 7.1 Hz, 1H), 0.96 (d, *J* = 6.1 Hz, 1H) ppm; **^{13}C NMR (101 MHz, Acetone-d6)** δ 174.35, 172.12, 172.02, 171.84, 170.62, 170.24, 159.11, 157.56, 156.94, 139.01, 138.89, 135.26, 133.00, 129.25 (overlap 2 aromatic C-H, phg), 128.46, 125.42, 117.61, 87.07, 81.89, 78.59, 58.20, 56.03, 55.19, 52.61, 43.91, 43.70, 43.46, 41.05, 38.28, 31.21, 30.38, 28.75, 28.13, 26.68, 24.40, 19.59, 18.35, 13.21, 12.60 ppm.

1.6. *cyclo*(phg- *iso*DGR-*k*) TFA salt (7)

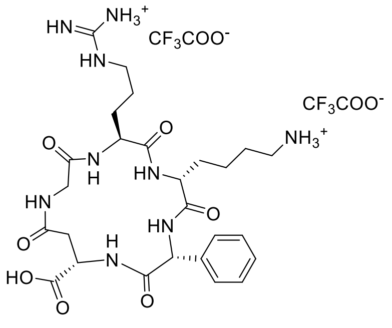

$C_{26}H_{39}N_9O_7$
MW = 589.6540 g/mol

TFA salt
$C_{30}H_{41}F_6N_9O_{11}$
MW = 817.7004 g/mol

The protected *c*[phg-*iso*Asp(OtBu)-Gly-Arg(Pbf)-*k*(Boc)] peptide (**6**) (24 mg, 0.024 mmol, 1 equiv.) was dissolved in 6 mL of a cleavage cocktail TFA/EDT/TIS/water (95:2:2:1) at 0°C. The reaction mixture was allowed to reach room temperature and stirred for 3 hours. Upon completion, the solvent was removed under reduced pressure. To residue was washed three times with toluene, followed by Et_2O (3x), with solvent removal under vacuum after each addition. The crude product obtained was purified by RP-HPLC [Gradient (15 ml/min flux): from 95% (H_2O + 10% CH_3CN) / 5% (CH_3CN + 10% H_2O + 0,1% HCOOH) to 50% (CH_3CN + 10% H_2O + 0,1% HCOOH) in 20 min, t_R = 9.40 min]. Appropriate fractions were combined and concentrated under reduced pressure, followed by freeze-drying to afford **7** a yellow solid (10.9 mg, 77%).

LC-MS (ESI+): *m/z* calc. for $[C_{26}H_{39}N_9O_7 + H]^+$ = 590.3045, found: 590.43; **^{1}H NMR (400 MHz, D_2O)** δ 8.46 (s, 2H), 7.64 (d, *J* = 6.7 Hz, 2H), 7.57-7.48 (m, 3H), 5.73 (s, 1H), 4.55 (d, *J* = 8.5 Hz, 1H), 4.49 (dd, *J* = 10.0, 3.3 Hz, 1H), 4.23-4.19 (m, 1H), 4.17 (d, *J* = 5.8 Hz, 1H), 3.81 (d, *J* = 17 Hz, 2H), 3.34 – 3.18 (m, 2H), 3.02 – 2.89 (m, 2H), 2.83 (dd, *J* = 14.1, 2.4 Hz, 1H), 2.61 – 2.49 (m, 1H), 2.05-1.98 (m, 1H), 1.86 (dd, *J* = 15.0, 7.3 Hz, 2H), 1.80 – 1.51 (m, 6H), 1.43-1.32 (m, 2H) ppm; **^{13}C NMR (101 MHz, D_2O)** δ 175.24, 173.68, 173.13, 171.52, 170.79, 156.79, 135.81, 129.36, 129.09, 128.45, 57.48, 55.20, 53.47, 52.73, 46.70, 41.41, 40.53, 39.00, 29.15, 26.80, 26.07, 24.47, 22.18 ppm.

1.7. *cyclo*[phg-*iso*DGR-*k*]-PEG4-N_3 (8)

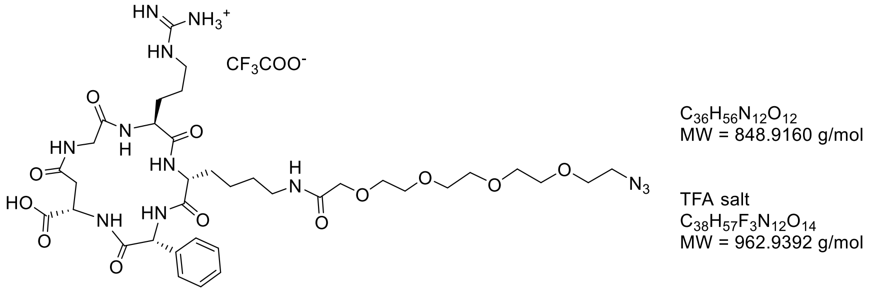

A solution of previously synthesized[1–3] N_3-PEG4-CH_2COOH (31.89 mg, 0.115 mmol, 1 equiv.) in DMF (1 mL) was cooled in the ice bath and purged with N_2. DIPEA (60.1 µL, 0.345 mmol, 3 equiv.), COMU (59.10 mg, 0.138 mmol, 1.2 equiv.), and OXIMA (19.61 mg, 0.138 mmol, 1.2 equiv.) were added subsequently. The mixture was stirred for 10 min, after which a solution of *cyclo*(phg-*iso*DGR-*k*) (74 mg, 0.126 mmol, 1.1 equiv.) in DMF (1 mL) was added dropwise. The reaction mixture was allowed to reach room temperature and stirred overnight. After LC-MS showed the formation of the product with the desired mass m/z = 849.86 $[M+H^+]$, the reaction was stopped and concentrated under reduced pressure. The residue was dissolved in ACN/H_2O 1/1 and purified using RP-HPLC [Gradient (15 ml/min): from 100% (H_2O + 10% CH_3CN) to 100% (CH_3CN + 10% H_2O + 0,1% HCOOH) in 30 min]. Appropriate fractions were combined, concentrated, and freeze-dried for two days to afford **8** as a white solid (72.8 mg, 75%).

HRMS (ESI+): m/z calc. for $[C_{36}H_{57}N_{12}O_{12} + H]^+$ = 849.4174, found: 849.4207; **^{1}H-NMR (400 MHz, D_2O)** δ 8.48 (s, 1H) 7.63 (d, *J* = 6.8 Hz, 2H), 7.57 – 7.50 (m, 3H), 5.74 (s, 1H), 4.51 (dd, *J* = 11.4, 3.3 Hz, 1H), 4.47 (dd, *J* = 10.5, 3.6 Hz, 1H), 4.20 (d, *J* = 5.5 Hz, 1H), 4.17 (d, *J* = 3.4 Hz, 1H), 4.05 (d, *J* = 3.9 Hz, 2H), 3.84 - 3.80 (m, 1H), 3.76 – 3.70 (m, 14H), 3.51 – 3.48 (m, 2H), 3.28 (dd, *J* = 12.1, 6.6 Hz, 2H), 3.22 (t, *J* = 6.8 Hz, 2H), 2.83 (dd, *J* = 14.2, 3.4 Hz, 1H), 2.55 – 2.49 (m, 1H), 1.99 – 1.96 (m, 1H), 1.88 – 1.82 (m, 2H), 1.80 – 1.67 (m, 1H),

1.68 – 1.44 (m, 4H), 1.34 (m, 2H) ppm; **^{13}C NMR (101 MHz, D_2O)** δ 176.12, 175.21, 173.57, 173.23, 172.24, 171.42, 170.81, 156.74, 135.87, 129.36, 129.07, 128.39, 70.32, 69.66, 69.59, 69.55, 69.51, 69.46, 69.20, 57.35, 55.26, 53.65, 52.76, 50.16, 41.30, 40.55, 39.11, 38.63, 29.42, 27.82, 26.76, 24.48, 22.69 ppm.

1.8. *cyclo*(phg-*iso*DGR-*k*)-PEG4-non-cleavable-*SAR405838* (1)

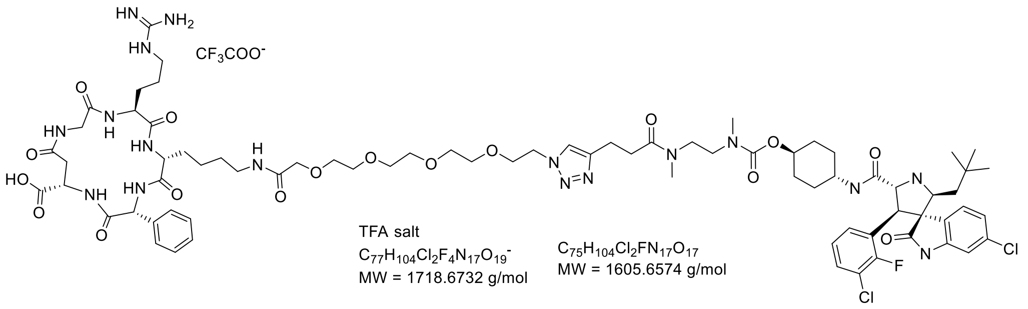

4-pentynamido-SAR405838 (**4**) (5 mg, 6.6 mmol, 1 equiv.) and *cyclo*(phg-*iso*DGR-*k*)-PEG4-N_3 (**8**) (5.84 mg, 6.9 mmol, 1.1 equiv.) were dissolved in a degassed 1:1 mixture of H_2O/DMF (1.3 mL) under N_2 atmosphere. Degassed aqueous solutions of $CuSO_4$ × $5H_2O$ (0.825 mg, 3.3 mmol, 0.5 equiv.) and sodium ascorbate (0.784 mg, 3.9 mmol, 0.6 equiv.) were added to the starting mixture at 0°C: the solution turned light yellow. The reaction was stirred for 16 h at room temperature, then the solvent was removed under vacuum, and the crude residue was purified by RP-HPLC [Gradient (15 ml/min flux): from 95% (H_2O + 10% CH_3CN) / 5% (CH_3CN + 10% H_2O + 0,1% HCOOH) to 70% (CH_3CN + 10% H_2O + 0,1% HCOOH) in 18 min., t_R = 13.9 min]. The purified product was then freeze-dried to give the desired conjugate **1** as a white solid (2.4 mg, 51% yield).

HRMS (ESI-): *m/z* calc. for $[C_{75}H_{104}Cl_2FN_{17}O_{17} - H]^-$ = 1602.7084 found: 1602.7087 $[C_{75}H_{104}Cl_2FN_{17}O_{17} - 2H]^{2-}$ = 800.8506 found: 800.8500. **HRMS (ESI+)**: *m/z* calc. for $[C_{75}H_{104}Cl_2FN_{17}O_{17} + 2H]^{2+}$ = 802.8651 found: 802.8649.

2. Biological analysis

1.9. Cell cultures

All cell lines were purchased from the American Type Culture Collection (ATCC, Manassas, VA, USA). The adenocarcinoma cell line MCF7 (code HTB-22) and the glioblastoma cell line U-87 MG (code HTB-14) were maintained in Eagle's Minimum Essential Medium (ThermoFisher Scientific, Waltham, MA, USA) and fetal bovine serum (FBS) to a final concentration of 10%. MCF7 cells were also supplemented with 0.01 mg/ml human recombinant insulin (Thermo Fisher Scientific, Waltham, MA, USA). The adenocarcinoma cell line SK-BR-3 (HTB-30) was maintained in McCoy's 5A Medium Modified (Thermo Fisher Scientific, Waltham, MA, USA) supplemented with 10% FBS. The non-small cell lung cancer cell line

1.10. Cell Proliferation Assay

U-87 MG glioblastoma cells were seeded at 1.6×10^3 cells per well in 96-well white polystyrene flat-bottom plates (Corning® CLS3903) in 90 µL of complete medium. Plates were incubated for 24 hours at 37 °C in a humidified atmosphere containing 5% CO_2. Tested compounds included SAR405838, *cyclo*(phg-*iso*DGR-k) (**7**), compound **4**, conjugate **1**, and co-treatments of SAR405838 with **7** in a 1:1 molar ratio. Stock solutions (10 mM) were diluted to treat cells with increasing concentrations of the compounds. Blank and vehicle (DMSO) controls were included. After compound addition, cells were incubated for 72 h. Cell viability was assessed using the CellTiter-Glo® 2.0 Assay (Cat# G7573, Promega, Madison, WI, USA) following the manufacturer's instructions. Luminescence was recorded using a VICTOR plate reader (PerkinElmer, Waltham, MA, USA), and relative cell viability was calculated as a percentage of untreated controls. IC_{50} values were determined using nonlinear regression in GraphPad Prism (v8.0.2).

1.11. α5β1 Integrin Expression by Flow Cytometry

α5β1 integrin surface expression was assessed in MCF7, SK-BR-3 and U-87 MG cell lines using flow cytometry. Cells were cultured under standard conditions and harvested at 70–80% confluency. 4×10^5 cells were transferred per well into a 96-well round-bottom plate and washed twice with FACS buffer (PBS1X containing Ca^{2+} and Mg^{2+}, 0.1% BSA, and 1 mM EDTA). Cells were incubated on ice for 1 hour with anti-α5β1 integrin primary antibody diluted 1:100 in FACS buffer (clone HA5, MAB1999, Merck KGaA, Darmstadt, Germany). After washing, cells were incubated with Alexa Fluor™ 647-conjugated goat anti-mouse IgG secondary antibody (A-21235; ThermoFisher Scientific, Waltham, MA, USA) diluted 1:4000

in FACS buffer for 20 minutes at 4 °C. Cells were then washed and fixed in 0.1% paraformaldehyde and analyzed by CytoFLEX flow cytometer platform (Beckman Coulter, Brea, CA, USA). Controls included unstained cells and cells incubated with secondary antibody only. Mean fluorescence intensity (MFI) values were compared across cell lines to quantify α5β1 integrin expression.

Table S2. α5β1 integrin expression across SK-BR-3, MCF-7, and U87-MG cell lines (triplicate).

Cell line	Replicate	% Positive Cells (P1)	Mean APC-A
SK-BR-3	1	23.06%	3,082.5
	2	23.17%	3,160.7
	3	23.16%	3,192.8
Average ± SD		23.13% ± 0.06%	3,145.3 ± 57.6
MCF-7	1	98.97%	16,765.4
	2	98.91%	15,566.0
	3	98.96%	15,749.8
Average ± SD		98.95% ± 0.03%	16,027.1 ± 655.0
U87-MG	1	99.66%	65,018.8
	2	99.82%	60,701.7
	3	99.92%	70,435.8
Average ± SD		99.80% ± 0.13%	65,385.4 ± 4,877.4

Figure S1 Mean fluorescence intensity (MFI, APC channel) of α5β1 integrin expression in MCF-7, SK-BR-3, and U87-MG cells. Data represent mean ± SD from three independent experiments (n = 3).

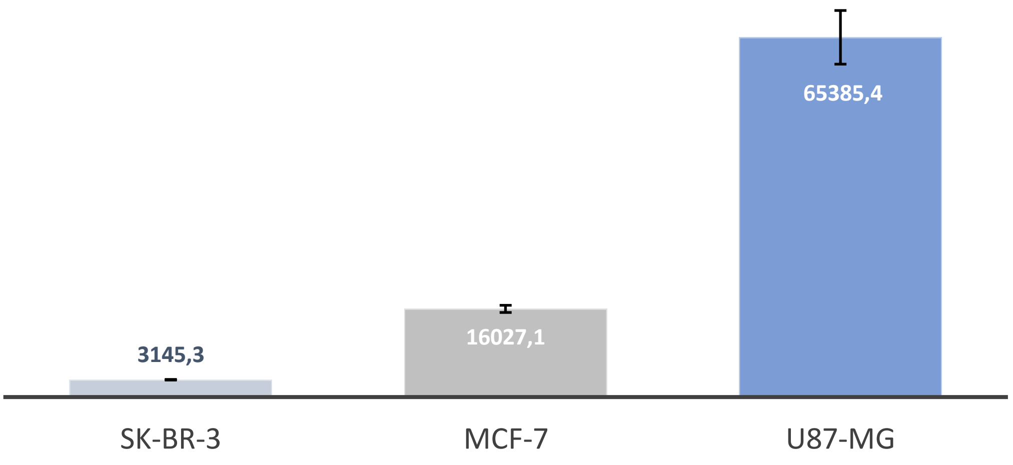

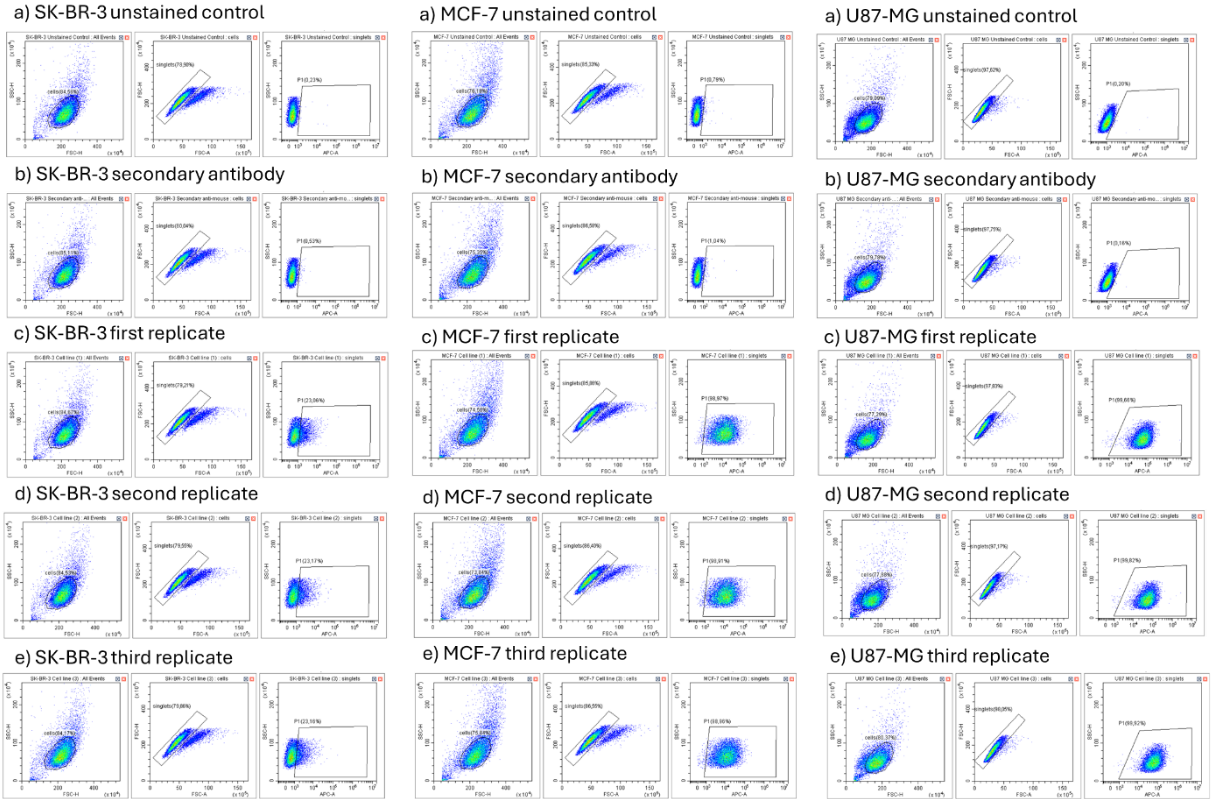

Figure S2 Flow cytometry analysis of α5β1 integrin surface expression in breast (SK-BR-3 and MCF-7) and glioblastoma (U87-MG) cell lines. Representative dot plots show (a) unstained controls, (b) secondary antibody controls, and (c–e) three independent biological replicates stained with anti-α5β1. Gating strategy included cells, singlets, and α5β1-positive populations (P1).

1.12. Western Blot Analysis

U-87 MG glioblastoma cells were seeded at 5 × 10^5 cells/well in 6-well plates and incubated for 24 h at 37 °C with 5% CO_2. Cells were treated with various concentrations (0.1, 1.0, 3.0, and 10.0 µM) of the conjugate **1**. Additional conditions included 3.0 µM SAR405838 and 10.0 µM of **7**. Untreated cells and cells treated with 0.1% DMSO were used as controls. After 24 h, cells were washed with PBS1X and lysed on ice with RIPA buffer (Merck KGaA, Darmstadt, Germany) supplemented with 25X protease and 10X phosphatase inhibitors. Lysates were incubated on ice for 15 minutes and centrifuged at 13,000 rpm for 30 seconds at 4 °C. Supernatants were collected and stored at -20 °C. Protein concentration was determined by the Bradford assay (Bio-Rad, Hercules, CA, USA) following the manufacturer's instructions. Protein samples (10 µg per condition) were prepared with NuPAGE sample buffer, DTT, and deionized water, then boiled at 90 °C for 5 minutes and loaded onto NuPAGE™ 4–12% Bis-Tris gels (Thermo Fisher Scientific, Waltham, MA, USA). Electrophoresis was performed at

90–150 V in 1X NuPAGE running buffer. Proteins were transferred onto 0.2 μm PVDF membranes using the Trans-Blot Turbo system (Bio-Rad, Hercules, CA, USA). Membranes were blocked for 15 minutes with EveryBlot Blocking Buffer (Bio-Rad, Hercules, CA, USA). After cutting, membranes were incubated overnight at 4 °C with the following primary antibodies, all diluted 1:1000 in blocking buffer: p53 (7F5, Cell Signaling Technology, Danvers, MA, USA). MDM2 (D1V2Z, #86934, Cell Signaling Technology, Danvers, MA, USA), p21 Waf1/Cip1 (12D1, #2947, Cell Signaling Technology, Danvers, MA, USA), and β-Actin (13E5, Cell Signaling Technology, Danvers, MA, USA) as loading control. After washing with 0.05% Tween-20 in PBS (PBS-T), membranes were incubated for 1 h at room temperature with HRP-conjugated anti-rabbit IgG secondary antibody (code #7074, Cell Signaling Technology, Danvers, MA, USA) diluted 1:3000. Detection was performed using Amersham ECL chemiluminescence reagent (Cytiva, MI, Italy), and Images were acquired using the Chemidoc system (Bio-Rad, Hercules, CA, USA).

1.13. Cell Cycle Analysis by Flow Cytometry

U-87 MG human glioblastoma cells were seeded at a density of 2×10^5 cells per well in 6-well plates and incubated for 24 hours at 37 °C in a humidified atmosphere containing 5% CO_2. Cells were treated with 10 μM of the following compounds: SAR405838, *cyclo(*phg-*iso*DGR-*k*) (5), and conjugate 1, and co-administration of 10 μM SAR405838 and 10 μM 5. Two controls were included: medium with 0.1% or 0.2% DMSO, and medium without FBS for 24 hours. After 72 h of incubation, cells were fixed and permeabilized using the Click-iT® EdU Flow Cytometry Assay Kit (C10633, ThermoFisher Scientific, Waltham, MA, USA) according to the manufacturer's protocol. DNA content was stained with FxCycle™ Violet (F10347, ThermoFisher Scientific, Waltham, MA, USA) in a saponin-based buffer. Samples were analyzed without washing by the CytoFLEX flow cytometer platform (Beckman Colter, Brea, CA, USA). Data were acquired and processed to assess phase distribution (G0/G1, S, G2/M phases), and at least 10,000 events were collected per sample.

Table S3. Flow cytometry analysis of the cell cycle in U87 MG cells treated with α5β1 ligand, SAR405838, conjugate **1**, and respective controls. Values indicate the percentage of cells in G0/G1, S, G2/M, and sub-G1 phases.

Condition	G0/G1 (%)	S (%)	G2/M (%)	sub G1 (%)
Control 1 (−)	0.00	0.00	0.00	100.00
Control 1 (+)	84.40	5.24	8.15	1.60
Control without FBS	84.09	5.10	8.65	1.51
Control DMSO (0.1 %)	82.02	5.94	10.00	1.40
Control DMSO (0.2 %)	82.32	6.01	9.77	1.21
10.0 µM SAR405838	85.07	2.90	7.42	3.91
10.0 µM ***c*****(phg-*****iso*****DGR-k) (7)**	81.53	7.26	9.06	1.24
10.0 µM conjugate **1**	41.00	9.31	35.90	12.01
10.0 µM SAR405838 + 10.0 µM **5**	86.99	3.09	5.93	2.49

Figure S2 Flow cytometry analysis of cell cycle distribution in U87-MG cells under different treatment conditions. Representative dot plots and histograms show the gating strategy (cells, singlets) and percentage of cells in G0/G1, S, G2/M, and sub-G1 phases. Treatments include a) and b) control (±FxCycle), c) serum starvation (0% FBS) d) and e) DMSO controls (0.1% and 0.2%), f) SAR405838 (10 μM), g) *cyclo*(*phg*-*iso*DGR-k) (**5**) (10 μM), h) combined treatment (SAR405838 and **5**, 10 μM each) and i) conjugate **1** (10 μM).

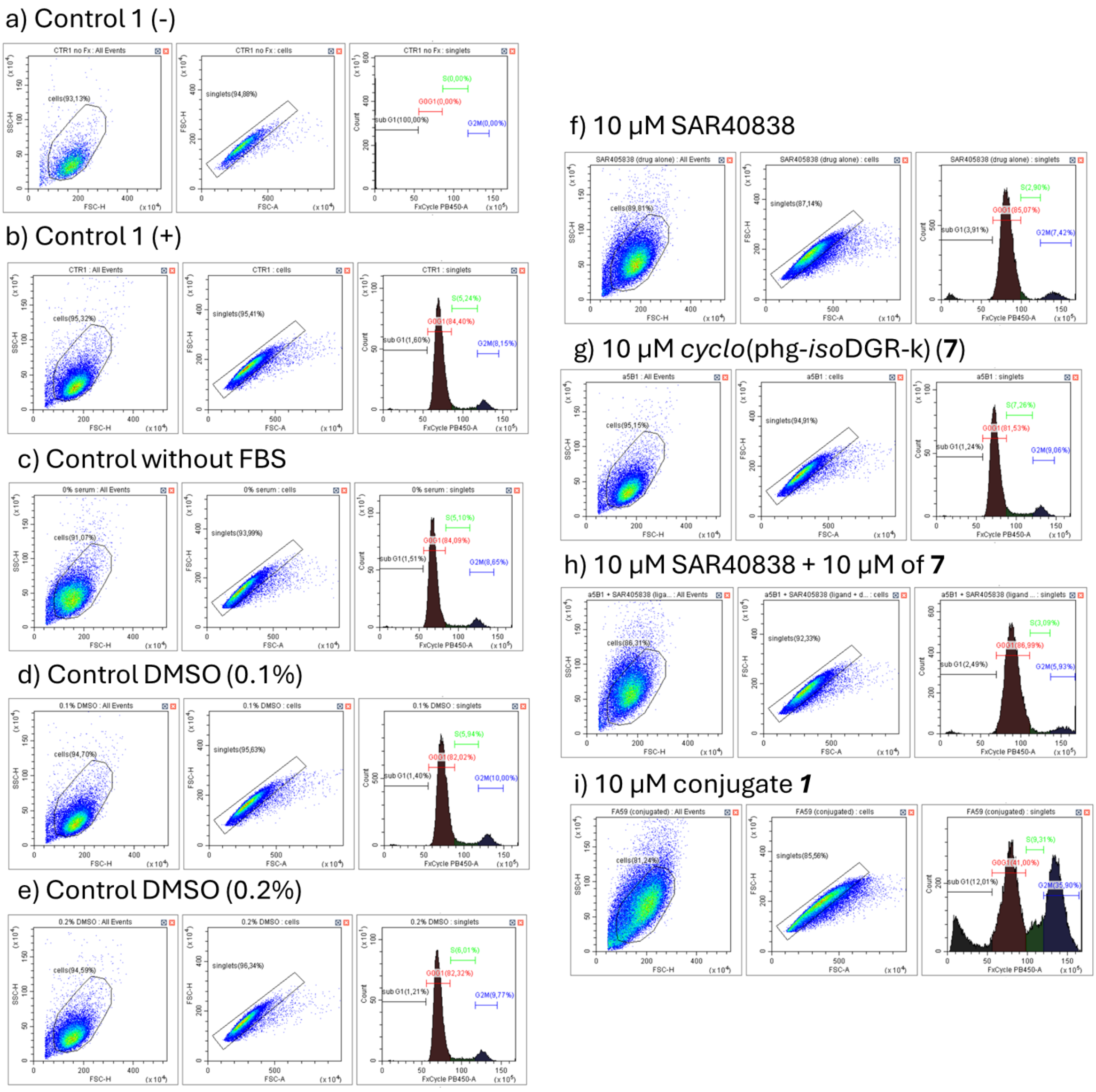

Analytical spectra

3. **HPLC traces and HRMS spectra**

1.14. 2’-(4-Nitrophenoxycarbonyl)-SAR405838 (2)

Gradient: 50% (H2O + 0.1% Formic acid) / 50% (CH_3CN + 0.1% Formic acid) to 95% (CH_3CN + 0.1% Formic acid) in 5 min, then isocratic for 15 min, t_R product: 6.49 min.

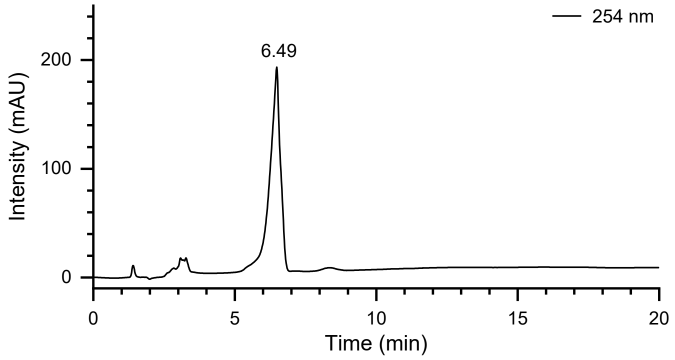

1.15. 4-pentynamido-SAR405838 (4)

Gradient: 50% (H2O + 0.1% Formic acid) / 50% (CH_3CN + 0.1% Formic acid) to 95% (CH_3CN + 0.1% Formic acid) in 5 min, then isocratic for 15 min, t_R product: 10.79 min

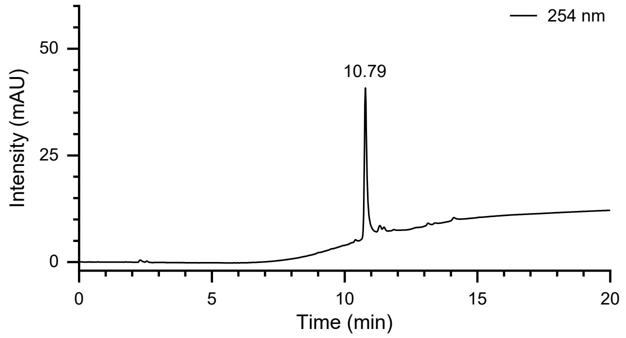

1.16. *cyclo*(phg-*iso*DGR-k)-non-cleavable-SAR405838 (1)

Gradient: 90%/10 to 10/90 in 12 minutes, then 10/90 for 3 min and 5/95 in 5 minutes (flux: 1 mL/min): t_r = 5.24 min

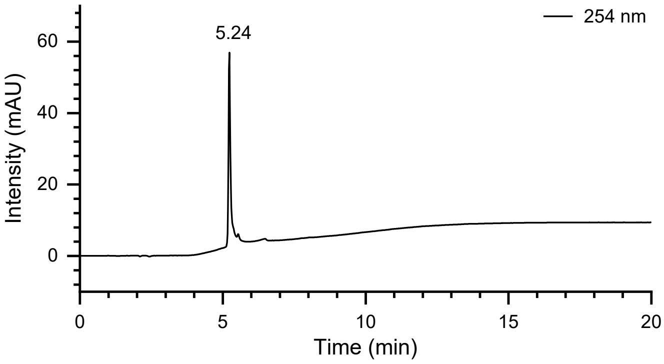

1.17. *cyclo*(phg-*iso*DGR-*k*)-PEG4-non-cleavable-SAR405838 (1)

HRMS (ESI-): *m/z* calc. for $[C_{75}H_{104}Cl_2FN_{17}O_{17} - H]^-$ = 1602.7084 found: 1602.7087 $[C_{75}H_{104}Cl_2FN_{17}O_{17} - 2H]^{2-}$ = 800.8506 found: 800.8500. **HRMS (ESI+)**: *m/z* calc. for $[C_{75}H_{104}Cl_2FN_{17}O_{17} + 2H]^{2+}$ = 802.8651 found: 802.8649.

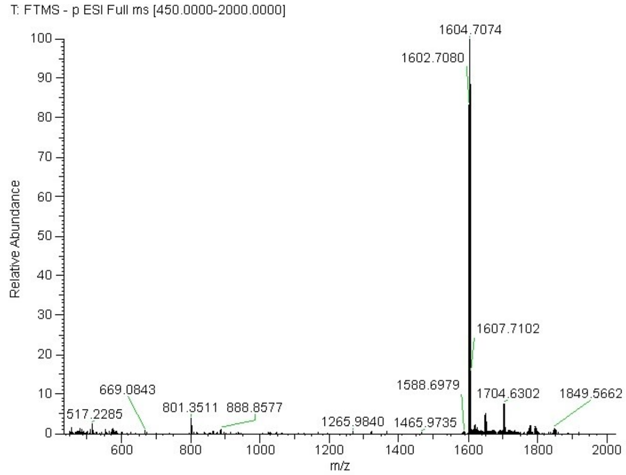

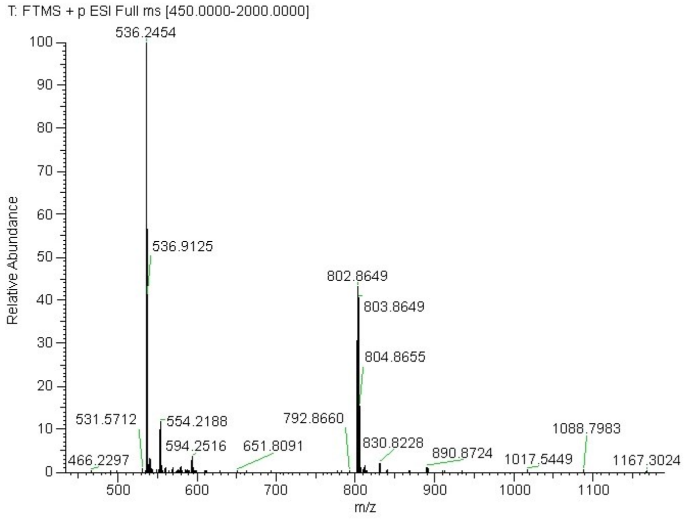

4. NMR spectra

1.18. *tert*-butyl methyl(2-(N-methylpent-4-ynamido) ethyl) carbamate (3)

^{1}H NMR (400 MHz, CD_2Cl_2)

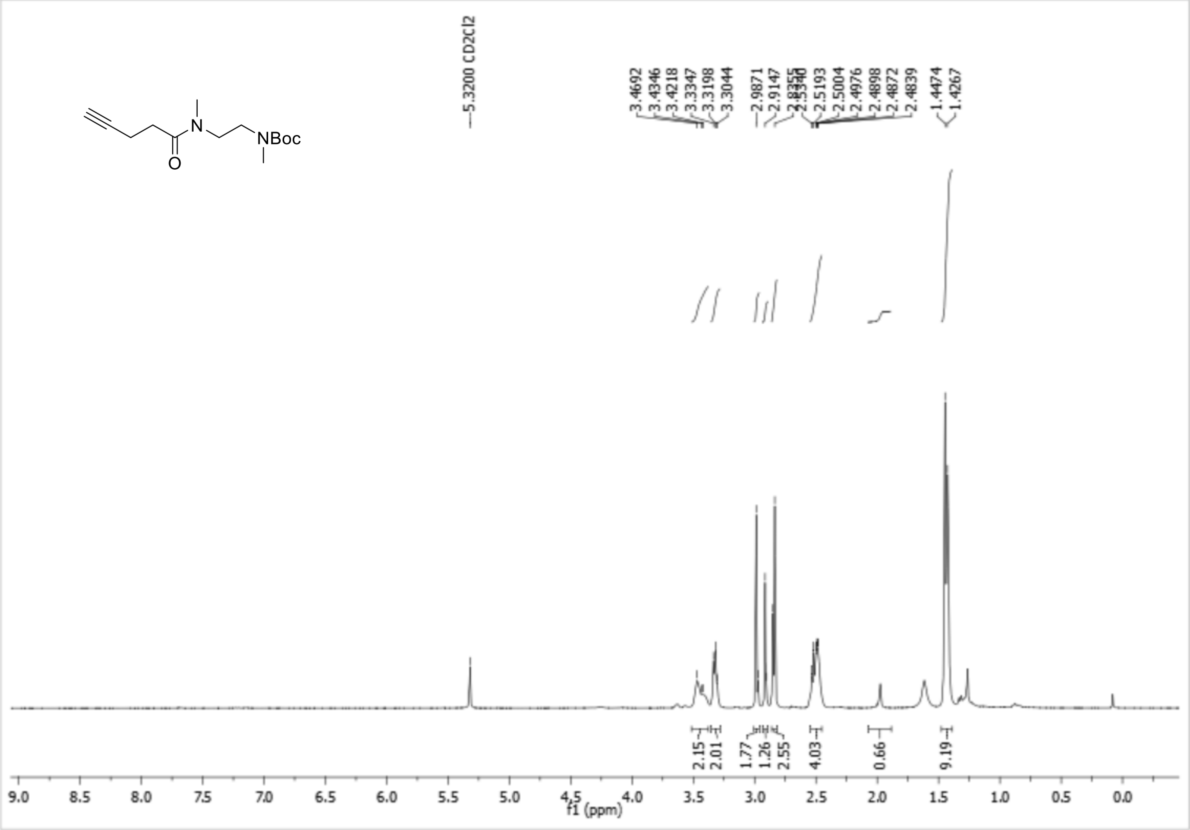

1.2. 4-pentynamido-SAR405838 (4)

a) ^{1}H NMR (400 MHz, Acetone-d$_6$)

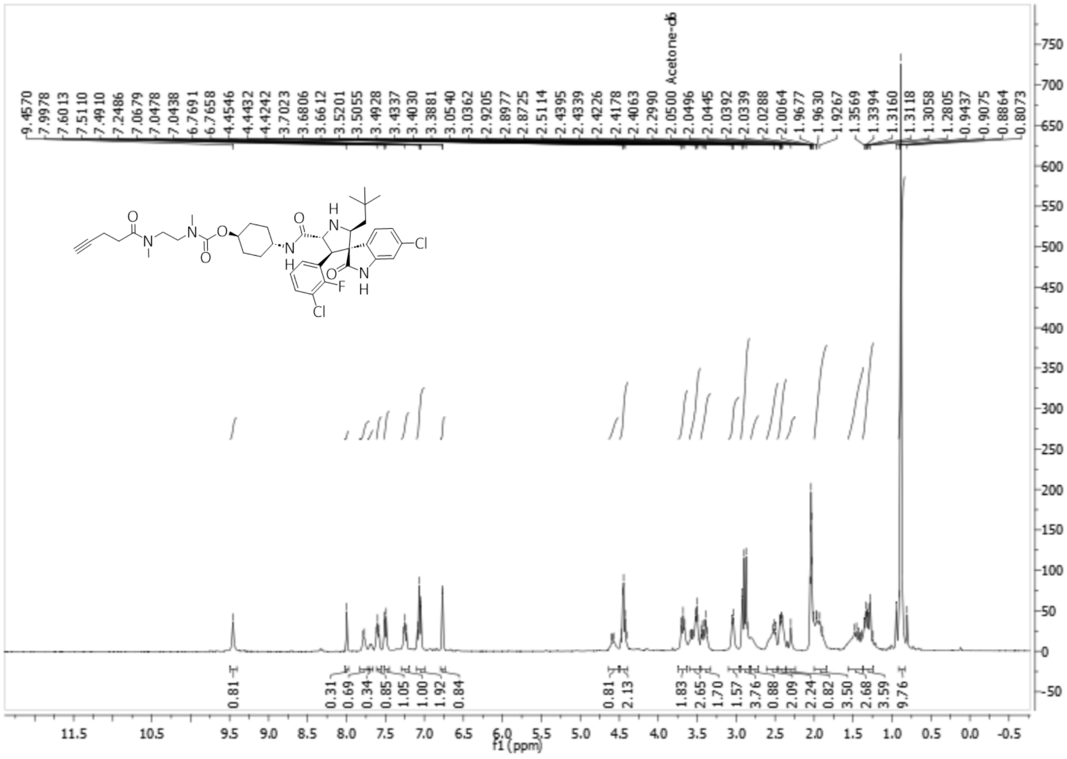

b) ^{13}C-NMR (101 MHz, Acetone-d$_6$)

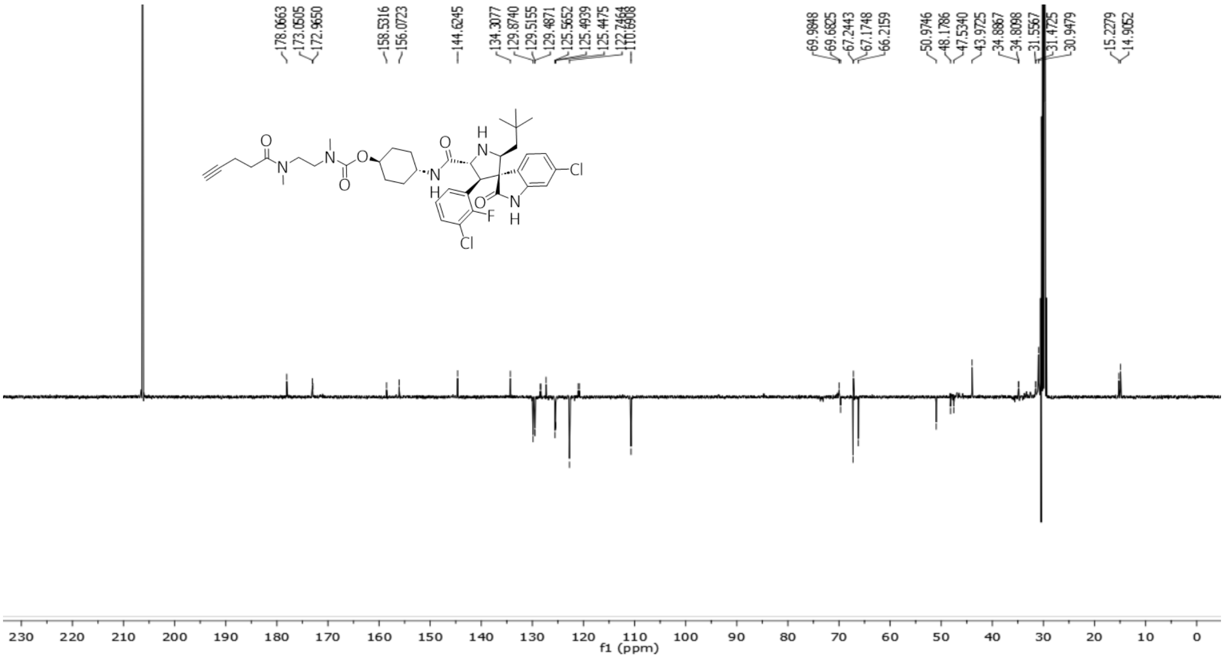

1.3.(H)-k(Boc)-phg--*iso*D(O^{t}Bu)-G-R(Pbf)-OH (5)

a) ^{1}H-NMR (DMSO-d_6)

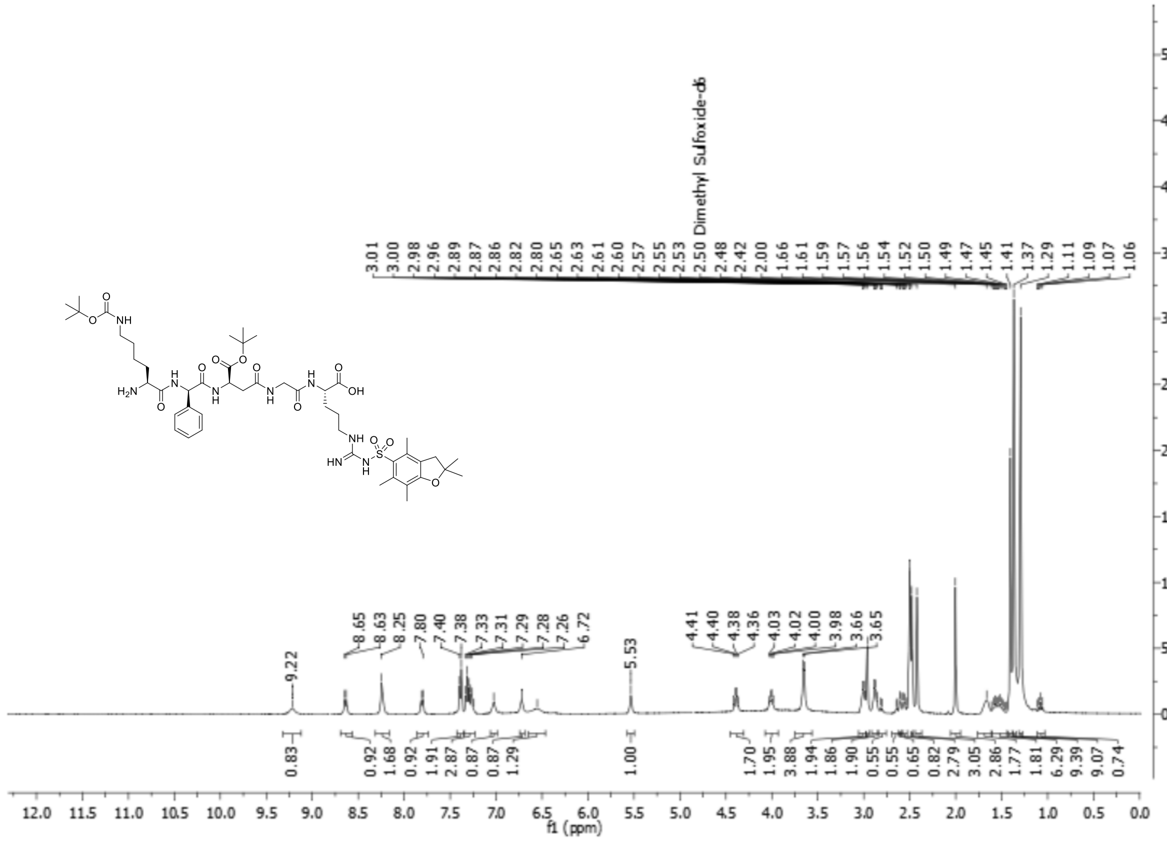

b) ^{13}C-NMR (DMSO-d_6)

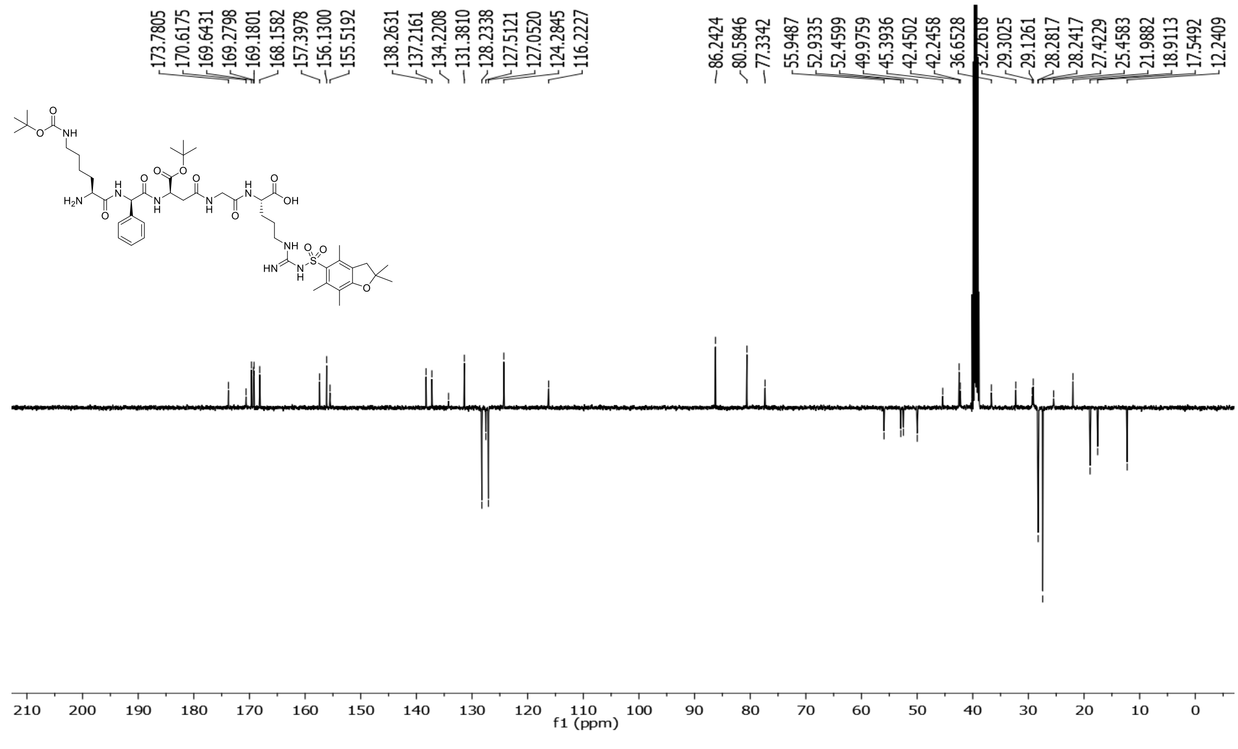

1.4. *Cyclo*[*k*(Boc)-phg--*iso*D(O^{t}Bu)-G-R(Pbf)] (6)

a) ^{1}H-NMR (acetone-d_6)

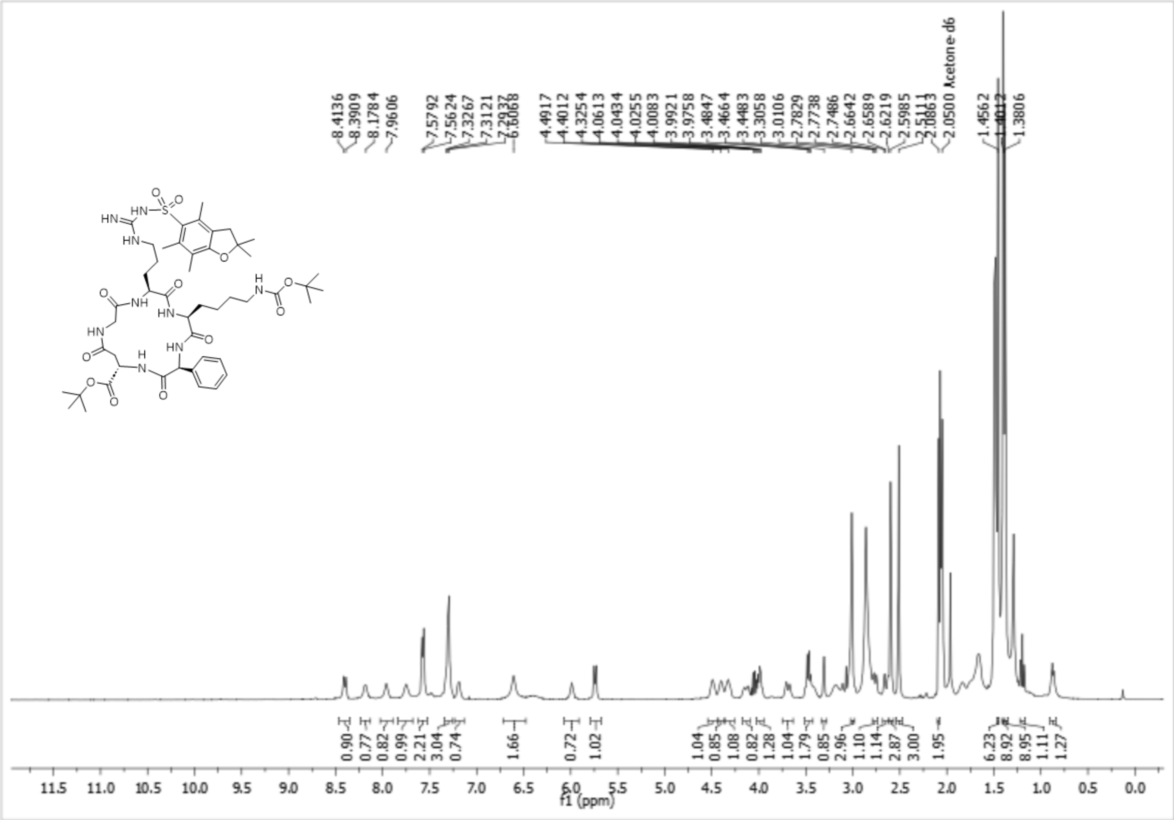

b) ^{13}C-NMR (acetone-d_6)

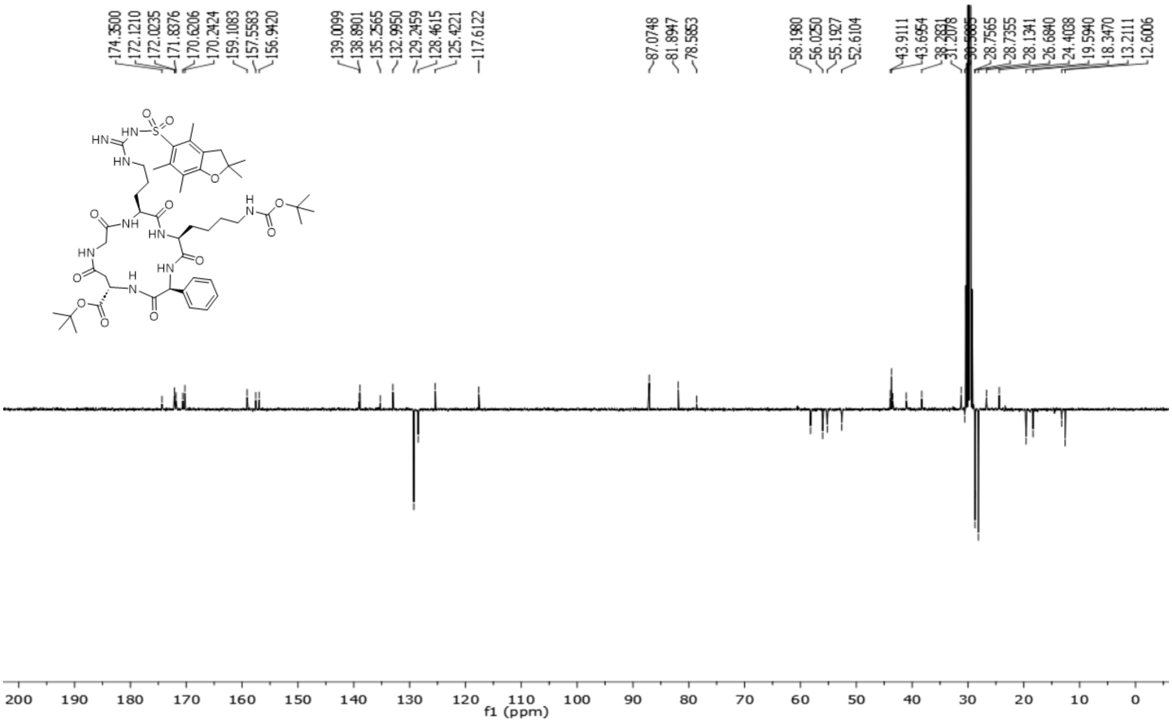

1.5. *Cyclo*[phg—*iso*DGR-*k*] (7)

a) ^{1}H-NMR (D_2O)

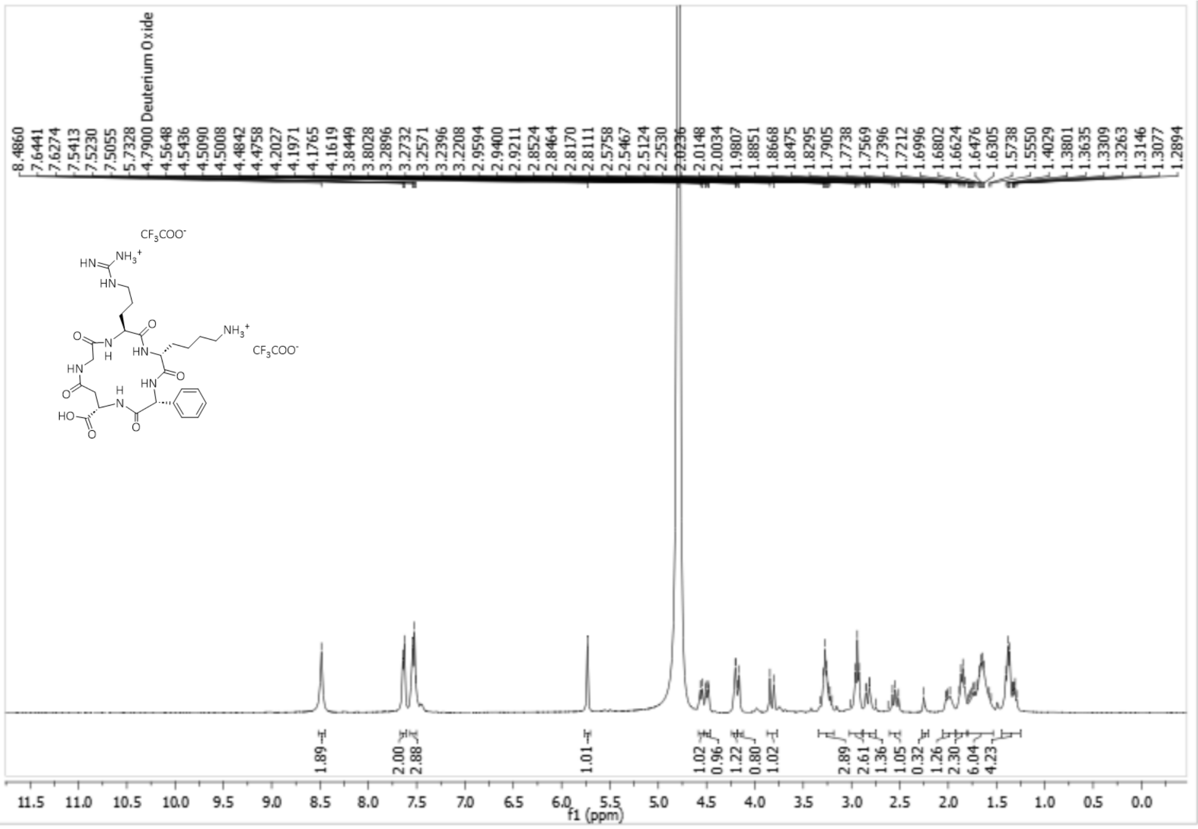

b) ^{13}C-NMR (D_2O)

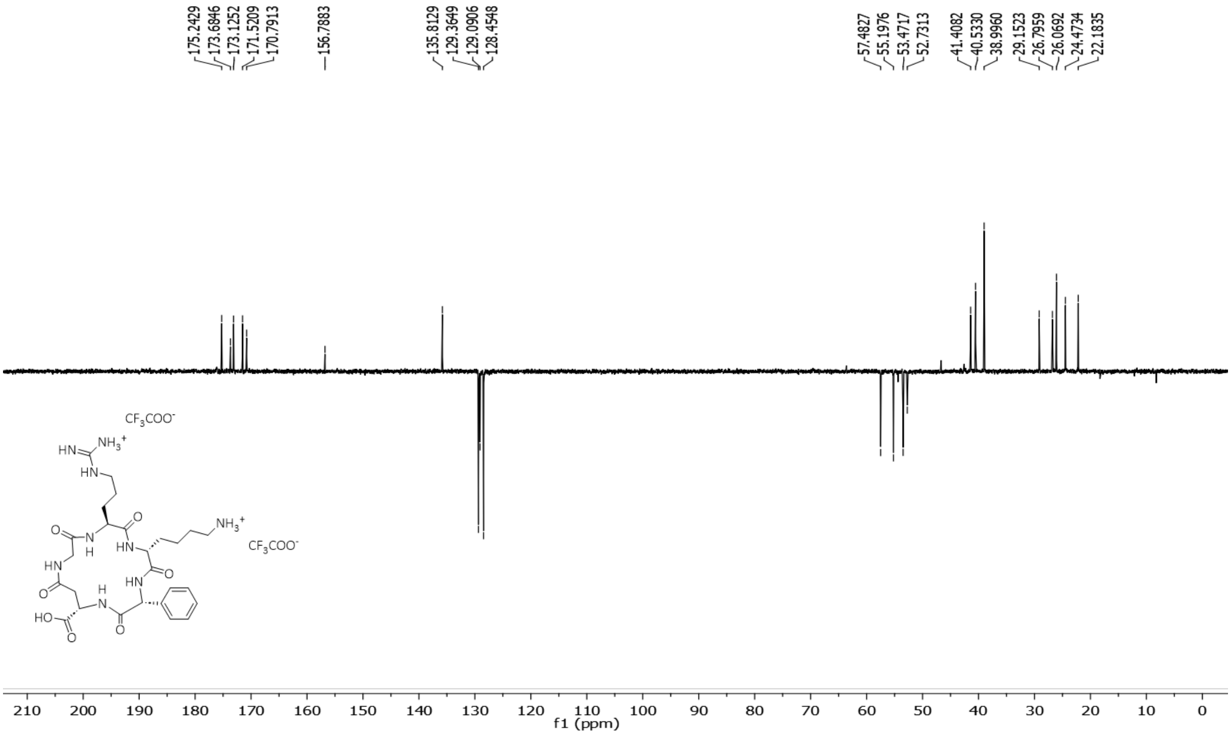

1.6. *Cyclo*[phg—*iso*DGR-*k*]-PEG4-N_3 (8)

a) ^{1}H-NMR (D_2O)

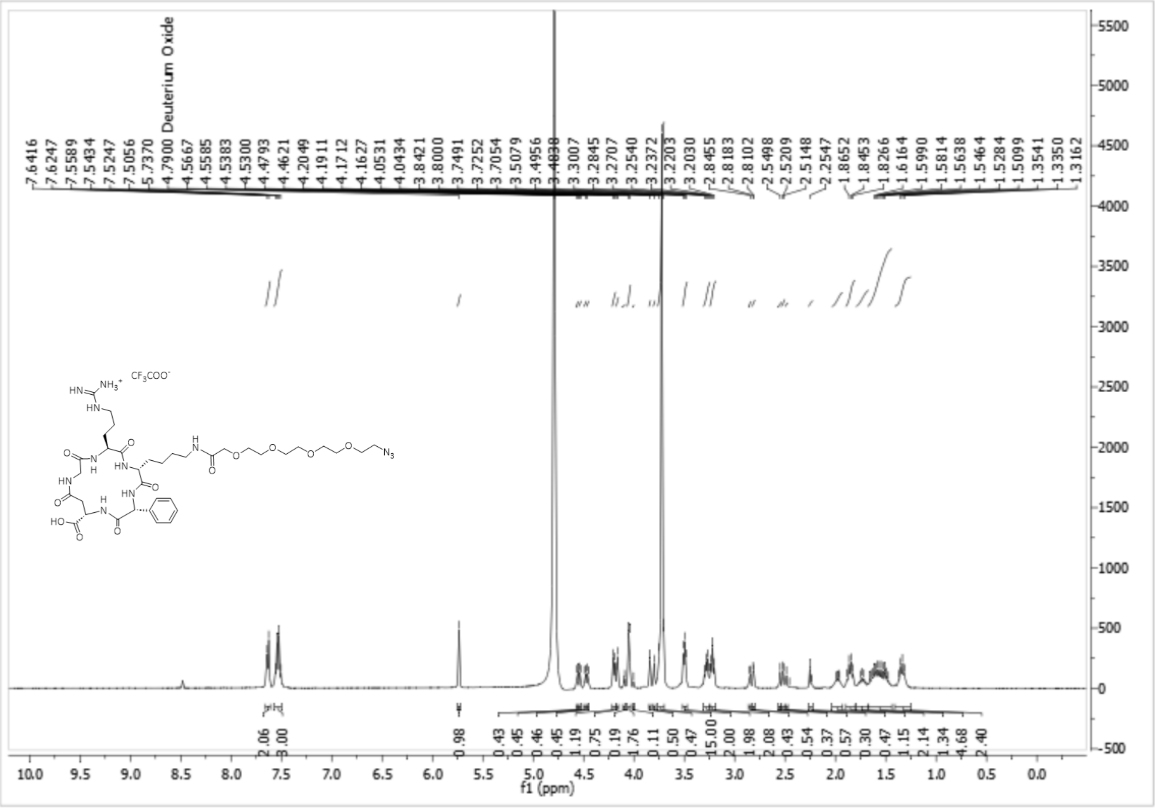

b) ^{13}C-NMR (D_2O)

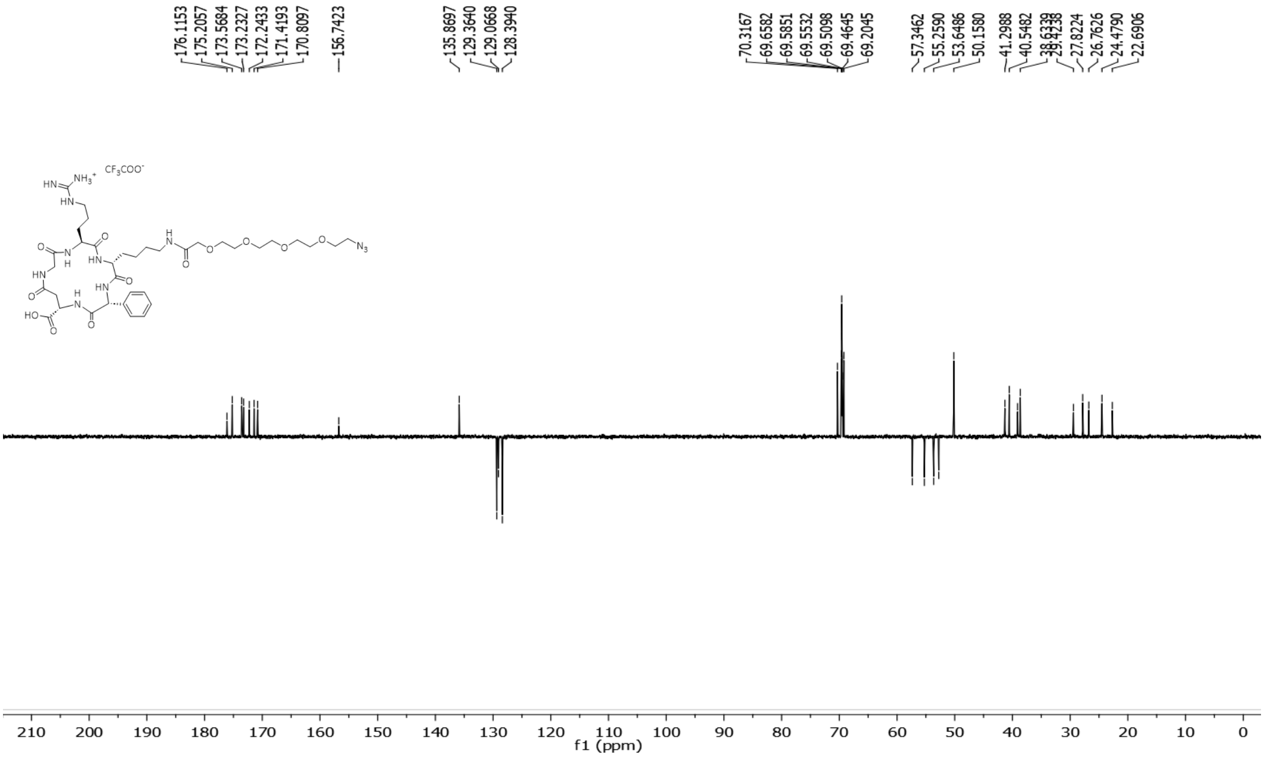

Supplementary references

(1) Bodero, L.; Parente, S.; Arrigoni, F.; Klimpel, A.; Neundorf, I.; Gazzola, S.; Piarulli, U. Synthesis and Biological Evaluation of an *Iso* DGR-Paclitaxel Conjugate Containing a Cell-Penetrating Peptide to Promote Cellular Uptake. *Eur J Org Chem* **2021**, *2021* (17), 2383–2387. https://doi.org/10.1002/ejoc.202100241.

(2) Borbély, A.; Figueras, E.; Martins, A.; Bodero, L.; Raposo Moreira Dias, A.; López Rivas, P.; Pina, A.; Arosio, D.; Gallinari, P.; Frese, M.; Steinkühler, C.; Gennari, C.; Piarulli, U.; Sewald, N. Conjugates of Cryptophycin and RGD or *Iso* DGR Peptidomimetics for Targeted Drug Delivery. *ChemistryOpen* **2019**, *8* (6), 737–742. https://doi.org/10.1002/open.201900110.

(3) Raposo Moreira Dias, A.; Pina, A.; Dean, A.; Lerchen, H.; Caruso, M.; Gasparri, F.; Fraietta, I.; Troiani, S.; Arosio, D.; Belvisi, L.; Pignataro, L.; Dal Corso, A.; Gennari, C. Neutrophil Elastase Promotes Linker Cleavage and Paclitaxel Release from an Integrin-Targeted Conjugate. *Chemistry A European J* **2019**, *25* (7), 1696–1700. https://doi.org/10.1002/chem.201805447.

(4) Bochen, A.; Marelli, U. K.; Otto, E.; Pallarola, D.; Mas-Moruno, C.; Di Leva, F. S.; Boehm, H.; Spatz, J. P.; Novellino, E.; Kessler, H.; Marinelli, L. Biselectivity of isoDGR Peptides for Fibronectin Binding Integrin Subtypes Α5β1 and Αvβ6: Conformational Control through Flanking Amino Acids. *J. Med. Chem.* **2013**, *56* (4), 1509–1519. https://doi.org/10.1021/jm301221x.

(5) Fields, G. B.; Noble, R. L. Solid Phase Peptide Synthesis Utilizing 9-fluorenylmethoxycarbonyl Amino Acids. *International Journal of Peptide and Protein Research* **1990**, *35* (3), 161–214. https://doi.org/10.1111/j.1399-3011.1990.tb00939.x.
